# Lipidome and Transcriptome Profiling of Pneumolysin Intoxication Identifies Networks Involved in Statin-Conferred Protection of Airway Epithelial Cells

**DOI:** 10.1038/srep10624

**Published:** 2015-05-29

**Authors:** Sarah Statt, Jhen-Wei Ruan, Chih-Ting Huang, Reen Wu, Cheng-Yuan Kao

**Affiliations:** 1 Center for Comparative Respiratory Biology and Medicine, University of California at Davis, Davis 95616 California; 2 Immunology Research Center, National Health Research Institutes, Zhunan, Miaoli 35053, Taiwan; 3 Research and Development Center for Immunology, China Medical University, Taichung 40402, Taiwan

## Abstract

Pneumonia remains one of the leading causes of death in both adults and children worldwide. Despite the adoption of a wide variety of therapeutics, the mortality from community-acquired pneumonia has remained relatively constant. Although viral and fungal acute airway infections can result in pneumonia, bacteria are the most common cause of community-acquired pneumonia, with *Streptococcus pneumoniae* isolated in nearly 50% of cases. Pneumolysin is a cholesterol-dependent cytolysin or pore-forming toxin produced by *Streptococcus pneumonia* and has been shown to play a critical role in bacterial pathogenesis. Airway epithelium is the initial site of many bacterial contacts and its barrier and mucosal immunity functions are central to infectious lung diseases. In our studies, we have shown that the prior exposure to statins confers significant resistance of airway epithelial cells to the cytotoxicity of pneumolysin. We decided to take this study one step further, assessing changes in both the transcriptome and lipidome of human airway epithelial cells exposed to toxin, statin or both. Our current work provides the first global view in human airway epithelial cells of both the transcriptome and the lipid interactions that result in cellular protection from pneumolysin.

Due to the constant exposure to the outside world, the lung presents a prime target for bacterial and viral infections. For the normal healthy adult airway, mucociliary clearance and acute immune responses usually work well enough to decrease the incidence of infection. Yet, in the compromised airway, such as seen in asthmatics, this is insufficient^1^. One of the most frequent bacterial infections found in asthmatics is *Streptococcus pneumoniae*, or pneumococcus^2^. This bacterial infection is also found in the general public and is the most common cause of bacterial pneumonia, as well as the root cause of some cases of bacterial meningitis, bacteremia, and otitis media^3^. *S. pneumoniae* infection is associated with both high morbidity and high mortality rates^3^,^4^. Although exact statistics are hard to determine, the World Health Organization estimates that 1.6 million deaths were caused by *S. pneumoniae* infection in 2008 (world-wide), with 333,000 to 529,000 of these occurring in children younger than 5 years^5^,^6^.

Pneumococcus is a polysaccharide capsulated gram-positive bacteria and more than 90 serotypes have been identified that vary in virulence and drug resistance^3^,^4^. Like many other bacteria, *S. pneumoniae* secretes pore-forming proteins during infection that aid in bacterial colonization. This pore-forming toxin, named pneumolysin (PLY), is a member of the cholesterol-dependent cytolysins (CDC) family secreted predominately in gram-positive bacterial pathogens^7^. Pneumolysin is secreted as a soluble monomer, which upon binding to host cell membranes via cholesterol, can oligomerize to form β-barrel pores and enter cells^8^,^9^. CDC infection is quite complicated, resulting in responses not only at the cellular membrane but also at many organelles inside the cell. Once the membrane is damaged, the cell works to repair the pore, removing the lesion in order to maintain its viability^10^. Yet, eventually the damage overwhelms the cell, ultimately resulting in cell death.

Statins are competitive inhibitors of 3-hydroxy 3-methylglutaryl coenzyme A (HMG-CoA) reductase, a key enzyme regulating cholesterol biosynthesis^11^. Due to their ability to inhibit cholesterol production, we began to investigate their use in protection from cholesterol-dependent cytolysins. More than 20 independent clinical epidemiological studies have been completed, suggesting that statins have a strong beneficial effect against pneumonia and sepsis related mortality^12^. Yet, statins’ beneficial role is still considered controversial and additional studies have suggested these so-called protective effects are due to misinterpretation of data, with one study actually showing an increase in infections in statin users^13^. In contrast, laboratory studies using animal models of infection have shown protection against bacterial infections under statin use, mainly focusing on *Staphylococcus aureus*^14^,^15^,^16^. We decided to test whether these potentially protective statins dependent effects could occur in the airway epithelium, the main physiological target of bacterial infections. Indeed, we have found that statins pretreatment led to protection *in vitro* from pneumolysin cytotoxicity in human airway epithelial cells. To further understand the global response networks behind this protection, we characterized both the transcriptome and lipidome of human airway epithelial cells exposed to toxin, statins or both. Due to the fact that we were exposing human airway cells to statins, competitive inhibitors of 3-hydroxy 3-methylglutaryl coenzyme A (HMG-CoA) reductase, a key enzyme regulating cholesterol biosynthesis that results in changes to cholesterol dynamics, it was logical to assess changes in lipid composition^11^. Currently, there are only a limited amount of reports involving the lipidomic analysis of statins, with a majority carried out with human plasma samples^17^,^18^,^19^. This study is the first to analyze lipid profiles of both statin-treated and pneumolysin-treated human airway epithelial cells.

## Results

### Statin pretreatment led to global changes in plasma membrane lipid composition

**Figure 1:**
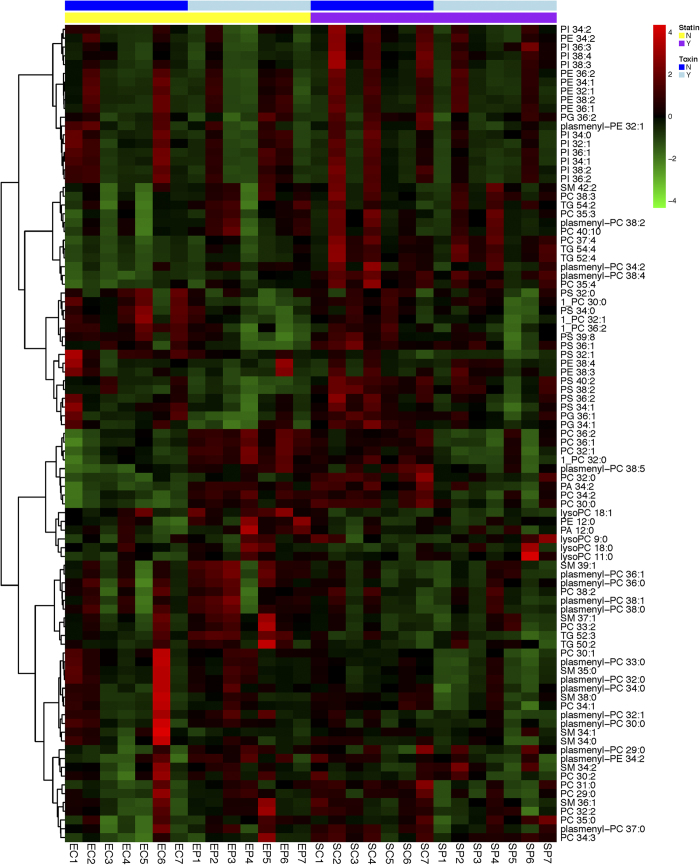
Global identification of lipid members in human bronchial cells (HBE1). Heatmap showing hierarchical clustering of 89 identified lipid members via mass spectrometry in the 4 classes of treatments: no treatment/vehicle control (EC), pneumolysin (400 ng/mL) alone for 4 hours (EP), simvastatin (1 μM) 24 hour treatment alone (SC) and both simvastatin (1 μM) 24 hour pretreatment and pneumolysin (400 ng/mL) for 4 hours (SP). Each experimental exposure was completed seven times in HBE1 cells and all independent samples are shown as z-score data. Intensities were normalized by class and underwent z scale centering by individual lipid. Clustering analysis was completed using Euclidean distance measures and the Ward algorithm.

**Figure 2:**
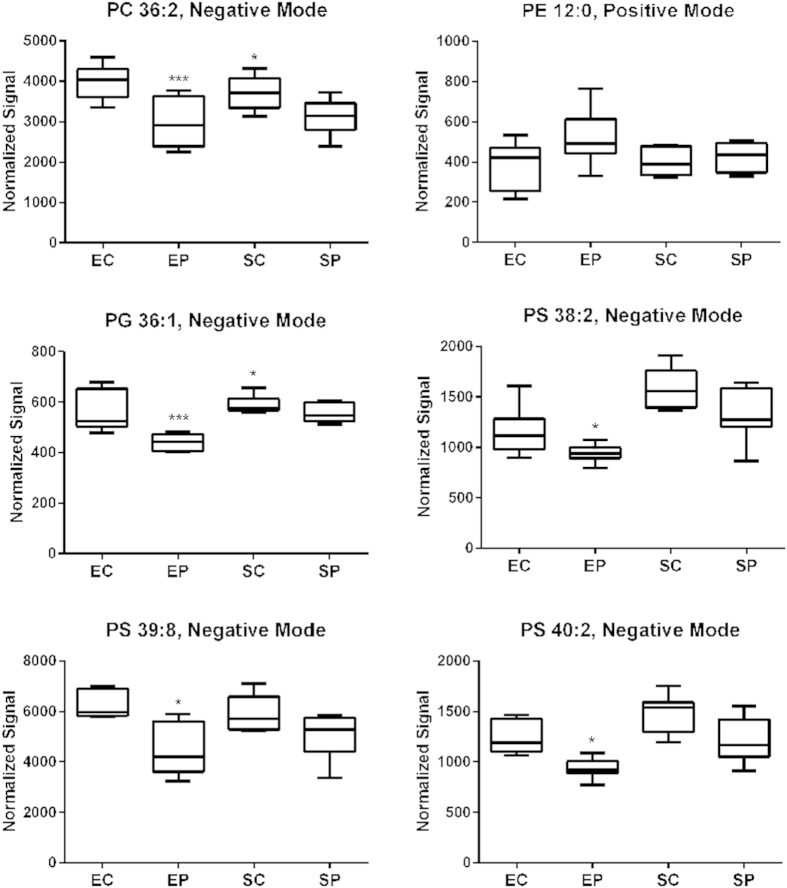
Pore-forming toxin exposure led to a decrease in lipid complexity, resulting in more saturated, short chain lipids. Six box plots characteristic of this trend are shown for the following lipids: PC 36:2, PE 12:0, PG 36:1, PS 38:2, PS 39:8 and PS 40:2. Human bronchial cells (HBE1) underwent the following treatments, for a total of seven replicates: no treatment/vehicle control (EC), pneumolysin (400 ng/mL) alone for 4 hours (EP), simvastatin (1 μM) 24 hour treatment alone (SC) and both simvastatin (1 μM) 24 hour pretreatment and pneumolysin (400 ng/mL) for 4 hours (SP). Intensities were normalized by class. Mean ion intensities with standard errors (boxes) and quartile ranges (whiskers) are shown. Positive mode and negative mode refer to the setting on the mass spectrometry under which this data was obtained. Statistically significant differences were assessed by one-way ANOVA with post hoc Dunnett’s correction and represented by asterisks as compared to Control.

**Figure 3:**
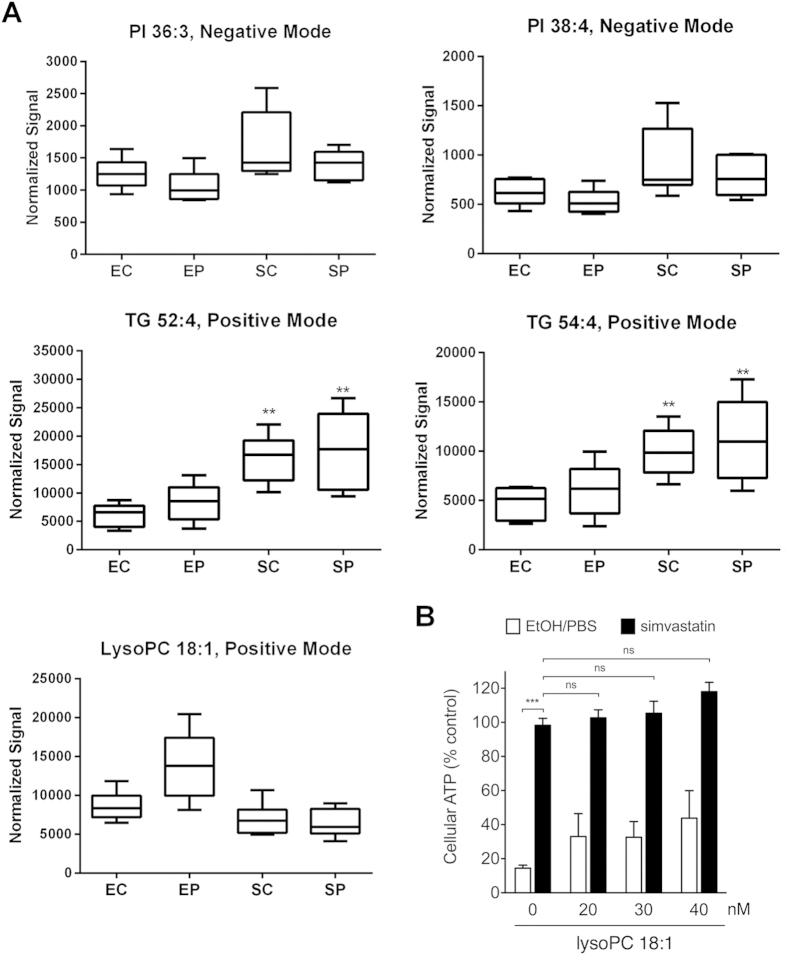
Statin pretreatment led to an increase in cell survival and lipid complexity, resulting in more unsaturated, long chain lipids. **A**.) Five box plots characteristic of this trend are shown for the following lipids: PI 36:3, PI 38:4, TG 52:4, TG 54:4 and lysoPC 18:1. Human bronchial cells (HBE1) underwent the following treatments, for a total of seven replicates: no treatment/vehicle control (EC), pneumolysin (400 ng/mL) alone for 4 hours (EP), simvastatin (1 μM) 24 hour treatment alone (SC) and both simvastatin (1 μM) 24 hour pretreatment and pneumolysin (400 ng/mL) for 4 hours (SP). Intensities were normalized by class. Mean ion intensities with standard errors (boxes) and quartile ranges (whiskers) are shown. Positive mode and negative mode refer to the setting on the mass spectrometry under which this data was obtained. Statistically significant differences were assessed by one-way ANOVA with post hoc Dunnett’s correction and represented by asterisks as compared to Control. **B**.) HBE1 cells were pretreated with simvastatin (100 nM) with or without indicated doses of lysoPC 18:1 for 24 hours and then challenged with pneumolysin (400 ng/mL) for 4 hours. Cellular ATP release assays were used to assess cell viability. Statistically significant differences between groups were assessed by one-way ANOVA with post hoc Tukey’s correction and represented by asterisks. Error bars represent S.E.M. of 3 experiments.

To help determine the molecular landscape characteristics of the affect of statins on pore-forming toxins, we decided to perform two types of global analysis on these lung epithelial cells. Since statins affect an acetyl-CoA driven pathway that plays a central role in lipid biosynthesis, we performed shotgun lipidomics on HBE1 cells grown under typical conditions, focusing on a rapid comparison of lipid profiles using nanoelectrospray ion trap tandem mass spectrometry (nanoESI-MS). We chose to use an immortalized cell line over primary lung epithelial cells due to the high technical noise present in this technique that would be hard to discern from biological variation. This method produced over 700 distinguishable ions per mass spectrum in positive mode and over 600 distinguishable ions per mass spectrum in negative mode. Although not all of these spectra could be identified due to the lack of liquid chromatography separation and subsequent lipid adduct ambiguity, 132 unique lipid structures plus 22 metabolites were found using an in-house LipidBlast database (Table ST1). Of these, 89 lipid structures were quantitated to determine altered lipids between the four treatment groups: EC (no treatment), EP (pore-forming toxin only), SC (statin only), and SP (both statin pretreatment and pore forming-toxin). This is shown in Table ST2 and Fig. 1. We found that 24 lipids were significantly changed in airway epithelial cells, using ANOVA and a P value cutoff of less than 0.01 (Table 1). Of the 89 quantifiable lipids, a majority of the changes were seen in major constituents of biological membranes: phosphatidic acids (PA), phosphatidylcholines (PC) and phosphatidylethanolamines (PE). Looking in more detail, PLY treatment led to a shift in lipid composition away from normal levels, concentrating on increasing saturated and shorter chained lipids (i.e. lysoPC 18:1, PE 12:0) while decreasing longer and unsaturated lipids (i.e. PC 36:2, PG 36:1, PS 38:2, PS 39:8, PS 40:2). In contrast, statin pretreatment led to a shift towards unsaturated lipids, as seen in PI 36:3, PI 38:4, and longer chained lipids (i.e. TG 52:4, TG 54:4). This lipid data is shown in figs 2 and 3.

### Addition of lysoPC 18:1 did not restore toxin dependent effects

C18:1 lysophosphatidylcholine or lysoPC(18:1) was increased during toxin treatment and statin pretreatment led to a statistically significant decrease, on par with normal levels of this lipid (Fig. 3). Lysophosphatidylcholines are derived from phosphatidylcholines by partial hydrolysis via phospholipase A2 enzymes^20^. Due to its commercial availability, we decided to investigate whether lysoPC(18:1) supplement can abrogate the protective effect of simvastatin. HBE1 cells were pretreated with simvastatin (100 nM) accompanied with various doses of lysoPC(18:1) for 24 hours, then treated with pneumolysin (400 ng/mL) for 4 hours. By ATP release assay, we found that lysoPC(18:1) supplement did not abrogate the simvastatin-mediated protection against pneumolysin (Fig. 3). Interestingly, lysoPC(18:1) treatment led to an increase in cell viability, although not statistically significant, in both the simvastatin supplemented and control cells. Additionally, we showed a similar affect in simvastatin treated cells using the MTS assay and this was included as supplemental figure S2.

### Transcriptome analysis indicated a strong role for early growth response genes

In order to better understand the cellular mechanism behind statin cellular protection, we completed transcriptome analysis. Lung epithelial cells from three patients with no known lung diseases were used in this study and grown in proliferating conditions. Primary cells were used in this experiment due to the low inherent technical variation present in mRNA-seq. As before, 24 hour pretreatment with simvastatin or mock control was followed by four hours of pneumolysin exposure or mock control. This resulted in four different classes from three different patients: vehicle control, only statin, only toxin, and both statin and toxin. In order to gain a better insight into the inherent nature of these twelve libraries, unsupervised clustering was done by way of multidimensional scaling or MDS plot. One of the main issues when working with primary human tissue samples is the source of noise due to biological variation. This was exemplified in the MDS plot, shown in figure S1; the twelve libraries separated out into three clusters based on their human sample identification and not into four clusters based on their treatment class. This indicated an inherent issue with the data; the variation between patients in each treatment class is high, reducing the power of this experiment and making statistical testing more difficult. Therefore, in order to compensate for this nuisance factor, and due to the fact that this is a paired study design, we can compare the treatment to the control for each set of lung epithelial cells, effectively subtracting out the baseline differences between the patients. Using this additive model and fitting a generalized linear model within the edgeR package, we were able to determine statistically significant genes. At a P value of 1% or less, the none versus toxin comparison had 430 significant transcripts, the none versus statin comparison had 318 significant transcripts and the none versus both statin and toxin comparison had 2924 significant transcripts. The top twenty genes ranked based on fold change over untreated cells for each of these comparisons are shown in table 2 and qPCR verification is shown in figure S3.

As was expected, members of the HMGR pathway were pulled out in the statin alone treated group, such as farnesyl diphosphate synthase (FDPS), 3-hydroxy-3-methylglutaryl-CoA (HMG-CoA) synthase 1 (HMGCS1) and acetyl-CoA carboxylase beta (ACACB). Interestingly, there were many genes in common between the toxin and statin alone treatments. Surprising to us, was the increase of both TNF and IL-8 in the statin alone treated group, at levels similar to those seen in the toxin alone treated group, implying a similar inflammatory response to statin exposure. In the toxin alone treated group, many of the top hits were transcription factors, such as EGR1 (Early growth response gene 1), EGR2 (Early growth response gene 2) and ATF3 (Activating transcription factor 3). EGR1 and EGR2 are members of the immediate early gene family and are induced by a variety of different stimulants: growth factors, neurotransmitters, hormones, stress and injury. Stress response genes were also found in the toxin alone treated group. ATF3 is a member of the cAMP responsive element-binding (CREB) protein family of transcription factors and involved in the cellular response to stress. CREBRF (CREB3 regulatory factor), a gene also up regulated in the toxin alone treated cells, acts as a negative regulator of the unfolded protein response (UPR), an endoplasmic reticulum stress response^21^. Notably, interleukin 20, as well as interleukin 8, were up regulated in both the toxin alone treated group and the combined treated group. Interleukin 20 has been shown to promote *S. aureus* infection, another pore-forming toxin producing bacteria, by down regulating IL-1β- and IL-17A-dependent pathways^22^. Most of the genes found in the both statin and toxin treated group are a combination of the two individual treatments. For example, the fold change associated with both IL-8 and TNF are equal to the addition of the levels of the two individual treatments. Of note is the presence of phospholipase A2, group III (PLA2G3) in the both treated group. Secreted phospholipase A2 members have a wide variety of biological functions, including generating free fatty acids and lysophospholipids, and play a strong protective role in host defense against bacteria and viruses^23^.

**Figure 4:**
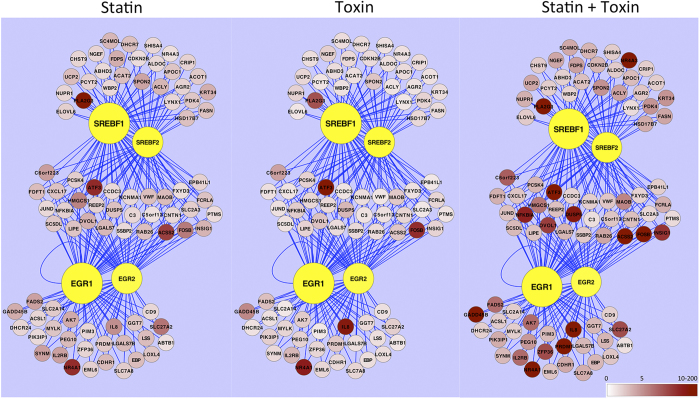
Pathway analysis showed a strong role for early response genes and sterol regulatory elements. Lung epithelial cells from three patients with no known lung diseases underwent the following treatments: no treatment/vehicle control, pneumolysin (400 ng/mL) alone for 4 hours, simvastatin (1 μM) 24 hour treatment alone and both simvastatin (1 μM) 24 hour pretreatment and pneumolysin (400 ng/mL) for 4 hours. Iregulon cytoscape plugin was used to interrogate the proximal promoter (500 bp within the TSS) of statistically significant transcripts (p value adjusted for false discovery by Benjamini–Hochberg cutoff of 0.1 or less) upregulated in response to treatment as compared to no treatment. EGR1 (Early growth response gene 1), EGR2 (Early growth response gene 2), SREBP1 (Sterol regulatory element-binding transcription factor 1) and SREBP2 (Sterol regulatory element-binding transcription factor 2) were all found to have putative binding sites to all transcripts shown (pink nodes). Normalized mRNA-seq expression levels, averaged across all three patients, are shown via a pink to red gradient for all transcripts as fold change values, comparing treatment indicated to no treatment/vehicle controls.

Interestingly, many of the genes regulated by EGR1 and EGR2 are also downstream of SREBP1 (Sterol regulatory element-binding transcription factor 1) and SREBP2 (Sterol regulatory element-binding transcription factor 2). Sterol regulatory element-binding proteins are a family of transcription factors involved in both lipogenesis and cholesterol homeostasis by controlling the expression of various enzymes required for cholesterol, fatty acid, triacylglycerol and phospholipid synthesis^24^. SREBPs are directly regulated by cholesterol levels and upon sensing a decrease, SREBPs are cleaved from the endoplasmic reticulum and allowed to translocate into the nucleus^24^. Through searching for transcription factor binding sites within 500 bp of the transcriptional start site (TSS) of a list of up regulated genes (at a P value of 1% or less) from the Statin only treated group, 67 binding domain motifs, with 47 unique transcription factors, were found. Of these, SREBP1 and SREBP2 were pulled out at the top of the list. This was completed using a plugin for cytoscape called iRegulon^25^ and the output is included in the supplemental as table ST4. We combined genes regulated by SREBP1/SREBP2 and EGR1/EGR2 as well as fold change expression values for all three treatment groups compared to untreated controls and visualized this data in fig. 4.

### Cluster analysis results reinforced the involvement of fatty acid metabolism

A common issue with genome wide RNA expression analysis involves interpreting the data in order to gain biological insights. Gene Set Enrichment Analysis (GSEA) helps in finding ways to solve this computational pigeonhole by providing an easy to use program that aids in finding overall themes of any large dataset^26^. Created by the Broad Institute, GSEA takes advantage of the knowledge of the scientific community and looks at the data in regards to sets of related genes (gene sets or GS). Due to this grouping into clusters, an increase in statistical power is gained and potentially changes the outcome of an otherwise unfruitful experiment.

Looking at the comparisons, many gene sets are pulled out as being significantly enriched. In both the Gene Ontology (GO) and the Kyoto Encyclopedia of Genes and Genomes (KEGG) gene sets, none versus statin, toxin or both statin and toxin comparisons, many of the same gene sets are pulled out in all three cases. All three cases have quite a large number of hits when using the 25% FDR cutoff recommended for hypothesis generation by the GSEA authors. In the GO work, statin alone group has 48 gene sets, toxin alone group has 53 gene sets and the both treatment group has 46 gene sets. In the KEGG work, statin alone group has 49 gene sets, toxin alone group has 43 gene sets and the both treatment group has 44 gene sets. The top ten hits for each comparison from this analysis are shown in table 3 and table 4.

As expected, due to the changes seen in the lipidomics work, many pathways involving fatty acid metabolism were found in both GO and KEGG gene sets (i.e. GO gene set lipid metabolic process and KEGG gene set biosynthesis of unsaturated fatty acids). Interestingly, translation was exclusively found in the toxin GO analysis, implying a dependency on protein production. Similar to the individual gene analysis, the gene ontology analysis shows an inflammatory response in the statin treated group, as well as the toxin alone and the both treated groups. Ultimately, many of the same pathways are found in all three comparisons, making it hard to discern out the specific biological processes involved in each treatment. In order to assist with this task, we completed leading edge analysis within GSEA on all three comparisons in order to better understand the main genes at work in each case. This type of analysis focuses on genes that are present in a subset of gene sets (chosen by the user; we focused on gene sets with a FDR of 25% or less) and assists in pulling out key genes important in each comparison. Although again many of the same genes were pulled out in all three comparisons, there are a few notable differences. Various members of the phospholipase A2 (PLA2) superfamily were pulled out in the none versus statin comparison and in the none versus both comparison (PLA2G3, PLA2G6 and PLA2G10), as seen in the single gene analysis. Again, these are of particular importance since there is a considerable body of evidence supporting the antibacterial function of PLA2 family members. Although it was not a top gene hit, in the none versus both comparison, IL-6 was found in 6 gene sets. IL-6 plays an important role in host defense against bacterial infection; using IL-6 -/- mice, it has been shown that IL-6 is required for resistance against *S. pneumoniae*^27^. Pore-forming toxins result in a wide variety of cellular responses and GSEA was able to pick out some important pathways involved.

## Discussion

In the present study, we have undertaken the first of its kind to investigate the changes in both lipids and RNA expression in lung epithelial cells exposed to a pore-forming toxin. Based on our data showing a protective effect from statin-pretreatment (as seen in Fig. 3B), we included this treatment in our current study in order to better understand the complex cellular processes associated with statin treatment. Statins were originally discovered by Dr. Akira Endo in the 1970s as a molecule produced from the fungus *Penicillium citrinum* as a means of self-preservation from other microbes and shown to inhibit the enzyme HMG-CoA reductase, leading to a decrease in cholesterol production^28^. It has been known for years that bacterial-fungal communities create environmental conditions that assist in controlling the growth of other microbes. Furthermore, due to the fact that the pore-forming toxin of interest in this study, pneumolysin, is a cholesterol dependent cytolysin, it is logical to believe that statin pretreatment may have an effect on PLY toxicity.

There are many intrinsic cellular pathways that have been shown to provide protection to pore-forming toxin (PFT) cytotoxicity, including mitogen-activated protein kinase (MAPK), Unfolded Protein Response (UPR), autophagy and calcium-dependent endocytic pathways, although the molecular mechanisms of how these occur remains unclear. Other groups have shown that statins may trigger mevalonate-independent pathways partially via a calcium increase and p38 MAPK activation, which induces UPR and cytoprotection in RAW264.7 cells^29^. Further complicating the story is the involvement of various inflammatory molecules, such as tumor necrosis factor (TNF) and interleukin 8 (IL-8) in all three treated groups and interleukin 20 (IL-20) in the toxin treated groups. TNF is a pro-inflammatory cytokine leading to the activation of one if not all of three pathways: nuclear factor kappa-light-chain-enhancer of activated B cells (NF-kappaB) activation, activation of MAPK pathways and induction of apoptosis^30^. Potentially as a consequence of the activation of NF-kappaB, IL-8 production increased. IL-8 production has been previously seen in response to various bacterial toxins, such as Staphylococcal α toxin^31^, aerolysin^32^, pneumolysin^33^ and *Listeria monocytogenes* toxin listeriolysin O^34^. Statins themselves have been shown to have both proinflammatory and anti-inflammatory properties. It has been shown that lipophilic statins (i.e. fluvastatin, simvastatin, atorvastatin, and lovastatin) were able to induce proinflammatory responses in both LPS stimulation^35^ and *Mycobacterium tuberculosis* exposure^36^. Interleukin 20, a member of the interleukin-10 family of cytokines, is a pro-inflammatory cytokine that is associated with both psoriasis (skin inflammation) and atherosclerosis (a chronic inflammatory disease resulting in lipid deposits). Interestingly, IL-20 has been previously shown to play a rather negative role in host defense; infection with *Staphylococcus aureus* led to the early up regulation of IL-20 family members (IL-19, IL-20 and IL-24), inhibiting the local generation of IL-1β and IL-17A. This ultimately leads to an increase in infection severity and led the authors to hypothesize that *S. aureus* may express a virulence factor that induces this expression^22^. Our study is the first case showing this same up regulation of IL-20 in response to the pore-forming toxin from *S. pneumonia.* Note: IL-24 was also up regulated in any toxin-exposed cells but not at a level that was statistically significant. Statin co-treatment did not decrease the expression of these IL-20 family members and its protective effects must be due to a different mechanism.

Furthermore, a role for phospholipases in statin conferred cellular protection is hinted at in this dataset. A2 phospholipases are a class of enzymes that catalyzes the release of arachidonic acid and lysophospholipids from phospholipids. Arachidonic acid is then further modified by cyclooxygenases into eicosanoids, including prostaglandins and leukotrienes, which can be both anti-inflammatory and inflammatory mediators depending on other factors^37^. A2 phospholipases are expressed and released (only the secreted subtype) by many human immune cells: macrophages, monocytes, T cells, mast cell and neutrophils. Many different isoforms of phospholipases are increased in concentration in the blood of patients with inflammatory or autoimmune diseases^38^,^39^. In normal systems, secreted phospholipases have been shown to be important in fighting both viral and bacterial infections, of which group IIA is the best characterized. GIIA sPLA2 has displayed antibacterial activity towards Gram-positive bacteria including *S. aureus* and *L. monocytogenes*^40^,^41^. Although sPLA2-IIA was not pulled out in this dataset, it has been shown by another group to be induced by statins in a dose dependent manner^42^. Overexpression of sPLA2-IIA in transgenic mice leads to improved clearance of bacteria in the lung and a decrease in mortality from *Staphylococcus aureus* infection^43^. This function is calcium-dependent and negated in the presence of EGTA, which is consistent with our BAPTA-AM data^44^,^45^,^46^. It is thought that the antibacterial activity depends on the ability of PLA2 to hydrolyze the bacterial cell membrane, due to the highly charged surface in the case of sPLA2-IIA^40^. Other members of the phospholipase A2 family have been shown to have antibacterial activity, with GX family members (PLA2G10 is of this family) being the second most potent sPLA2 against gram-positive bacteria (sPLA2-IIA is the most potent)^41^.

Since statins are competitive inhibitors of a key enzyme regulating cholesterol biosynthesis, it was logical to investigate a potential global lipid metabolic shift. According to the LIPID Metabolites And Pathways Strategy (LIPID MAPS), there are 8 main categories of lipids: Fatty acyls/acids (FA), Glycerolipids (GL), Glycerophospholipids (GP), Sphingolipids (SP), Sterol lipids (ST), Prenol Lipids (PR), Saccharolipids (SL) and Polyketides (PK). A majority of the lipids found to be statistically significant in our dataset were members of the glycerophospholipids category. Glycerophospholipids are key components of the lipid bilayer of cells and the following types are found in biological membranes: phosphatidylcholine (PC or lecithin), phosphatidylethanolamine (PE), phosphatidylinositol (PI) and phosphatidylserine (PS). We have hypothesized a role of statins in membrane remodeling and the fact that we have pulled out key lipids in membrane structures reinforces this idea. Interestingly, PLY exposure to the cells alone led to a dramatic shift in the saturated PC lipid profile (top statistically significant hits: PC 32:1, PC 34:2, and PC 36:1), the most abundant lipid subtype found in the plasma membrane. We found that statins appear to affect other lipid subtypes, such as triglycerides (TG), in addition to PC lipids. TG 54:4, one of the statistically significant TG lipids from the statin treated group, has been shown by another group to be up regulated in response to inflammation in a mouse macrophage LPS induced inflammatory model and expression of this lipid did not change with statin treatment^47^. Another subtype unique to the airway epithelium was the change seen in phosphatidylinositol (PI) lipids. Mouse macrophages do not show any change in expression with statin treatment whereas our work shows an increase in both PI 36:3 and PI 38:4 in response to simvastatin treatment. This is not surprising, due to the fact that PI lipids are more common in the airway than in macrophages^48^. Similarly, in the mouse macrophage, they showed that statin treatment resulted in a blockade of the formation of sterol esters, pushing temporal changes in fatty acids in response to inflammation to other categories, such as glycerolipids, glycerophospholipids and/or sphingolipids, which were all late onset lipids^47^. Further reinforcing our hypothesis for a role of phospholipases in statin-dependent protection, this group also saw an increase in expression of various PLA2 family members^47^.

The lipid complexity was also affected by simvastatin. Under statin exposure, an increase in unsaturated PC and PI lipids was detected (i.e. PC 37:4 and PC 35:4). Chain length and unsaturation levels affect both membrane thickness and membrane fluidity, both of which can affect cellular membrane integrity and ultimately, host cellular defense^49^. Looking directly at our work with lysoPC(18:1), our results showing a slight increase in cell viability with lipid co-treatment are counter to our initial expectations that the reduced lysoPC(18:1) conferred the cellular protection against PLY. However, since lysoPC(18:1) is known to increase during the initial stages of apoptosis and this lipid was significantly decreased when simvastatin was given before toxin exposure, our results may indicate that the decrease of lysoPC(18:1) could be a downstream effect caused from reduced cell death triggered by PLY intoxication. This is due to the ability of lysoPC lipids to interact with various pro-apoptotic proteins of the *bcl-2* family, having been shown to decrease the amount of bid protein associated with mitochondria^50^,^51^,^52^. It will be interesting to see what role lipid dynamics play in cellular protection as this field advances with the advent of more commercially available lipids.

A possible explanation for the lipid metabolic and complexity shift under simvastatin exposure could be due to simvastatin-regulated genes. Comparing our dataset with another RNA-seq dataset involving the exposure of human liver cells (HepG2 cell line) to atorvastatin, using the same log2FC cutoff of 1.2 used by this group^53^, we found 22 transcripts in common between these two datasets. Many of the genes enriched, such as *mvd* and *hmgcs1*, are involved in mevalonate and cholesterol biosynthesis pathways. It is plausible that statins in general up-regulate mevalonate pathway genes to compensate for a reduced output from the mevalonate pathway in both cell types. On the other hand, the rest of the regulated genes could be potential unique statin-mediated response in either cell type.

Overall, we have shown a role for statins in protection against the negative effects of pore-forming toxins. Through the use of next-generation sequencing and lipidomics, we have provided global insights into how this protection may occur. Furthermore, our findings reinforce the idea that statin use may be potentially developed as an adjuvant therapy to pore-forming toxin-related bacterial infection in the lung.

## Methods

### Ethics Statement

Excised human airways were collected from the NIH-sponsored National Disease Research Interchange (NDRI, Philadelphia, PA) procurement through informed consent of the donors or next-of-kin. The investigators performing the experiments are not involved in the recruitment or direct contact with the donors and their relatives. In addition, the tissues were anonymized and the investigators have no access to the personal information of the donors. The use of cells isolated from those airways has been approved by Institute Human Subject Review Committee of University of California Davis. The methods were carried out in accordance with approved guidelines.

### Cell Culture and Reagents

Simvastatin was purchased from Sigma-Aldrich. C18:1 lysophosphatidylcholine was purchased from Cayman. ROCK inhibitor Y-27632 was purchased from Reagents Direct. 6xHis tag-fused pneumolysin was expressed in and purified from an Escherichia coli M15 (pREP4)^54^. Residual LPS was removed by passage over AffinityPak Detoxi-Gel (Endotoxin Removal Gel; Thermo Fisher Scientific) following the manufacturer’s instructions.

HBE1 cells, a human papillomavirus immortalized bronchial epithelial cell line and a gift from JR Yankaskas at University of North Carolina, were cultured using BEGM Bronchial Epithelial Cell Growth Medium (Lonza, Basel, Switzerland), minus the retinoic acid: Ham’s F12/Dulbeco’s Modified Eagle (DME) medium (1:1) supplemented with insulin (5 μg/mL), transferrin (5 μg/mL), epidermal growth factor (10 ng/mL), dexamethasone (0.1 μM), cholera toxin (10 ng/mL), bovine hypothalamus extract (15 μg/mL), and bovine serum albumin (0.5 mg/mL). Regarding Normal Human Bronchial Epithelial (NHBE) cells, protease-dissociated tracheal and primary bronchial epithelial cells were collected from tissues of patients without diagnosed lung-related diseases and plated in BEGM Bronchial Epithelial Cell Growth Medium (Lonza) minus the retinoic acid with the addition of 5 μM ROCK inhibitor. All cells were maintained at 37 °C in a humidified incubator with 5% CO2.

### Illumina library preparation/sequencing

NHBE cells were seeded at 5 × 10^5^ cells per well of 6-well plates and grown overnight. Three wells were used per treatment. In two days, cells were exposed to simvastatin (1 μM, Sigma-Aldrich) or vehicle control in BEGM medium for 24 hours. The exposed cells were then treated with pneumolysin (400 ng/mL) or mock control for 4 hours. This resulted in 4 different classes from three different patients: vehicle control (N), only statin (S), only toxin (T), and both statin and toxin (B). Total RNA was extracted and purified using RNeasy kit (Qiagen) per manufacturer’s instructions. The RNA integrity (RIN Score > 9.0) and quantity was determined on the Agilent 2100 Bioanalyzer (Agilent) using the RNA 6000 nano kit following manufacturer’s instructions. RNA samples were prepared by Beijing Genomics Institute using the Illumina TruSeq RNA sample preparation kit (Illumina). Briefly, total RNA (2 μg) was used for poly-A mRNA selection using oligodT magnetic beads, with two rounds of enrichment. The samples were then fragmented using both heat and high salt conditions. cDNA synthesis, multiplex indexing, and PCR amplification was then completed accordingly, resulting in a dsDNA library for each of the 12 samples. Indexed libraries were pooled in equal concentrations and run on a HiSeq2000 for SR50. HiSeq2000 reads were aligned to the Hg19 transcriptome using Burrows-Wheeler Transform allowing for 4% mismatch followed by conversion to SAM files using SAMtools^55^. Indexed samples were then merged at the SAM file step, resulting in 12 samples. The read counts were extracted with a perl script (provided upon request), and then imported into R. Data analysis was completed using edgeR^56^ and network figures were created using cytoscape^57^ with the iRegulon plugin^25^. The raw data files have been uploaded to NCBI, under the following bioproject ID: PRJNA277544.

### Gene Set Enrichment Analysis (GSEA)

GSEA was run using the graphical user interface javaGSEA desktop application, downloaded from the GSEA website ( http://www.broadinstitute.org/gsea). Aligned data was adjusted to HUGO gene name format in order to upload into GSEA, using raw reads as expression values. A class file was created to indicate the group of each of the 12 samples to the GSEA program.

### Lipidomics

HBE1 cells were seeded at 5 × 10^5^ cells per well of 6-well plates and grown overnight. Three wells were used per replicate of each treatment. In two days, cells were exposed to simvastatin (1 μM) or vehicle control in growth medium for 24 hours. After medium change, the pre-exposed cells were then treated with pneumolysin (400  ng/mL) or mock control for four hours. This resulted in 4 different classes with seven replicates per condition: vehicle control (EC), only statin (SC), only toxin (EP), and both statin and toxin (SP). Cells were washed three times with PBS and pelleted at 10 RCF for 10 minutes at 4 °C. The PBS was removed and the pellet was snap frozen and stored at −80 °C until used. Samples were thawed and extracted with MTBE as described previously^58^. Briefly, the vortexed/grinded homogenized sample (30 μL) was placed in a 1.5 mL vial. Cold methanol (225 μL) and MTBE (750 μL) were added to each sample. Samples were vortexed and shaken for six minutes at 4 °C. Distilled water was added to bring total volume to 1.2 mL and samples were centrifuged for 2 minutes at 14,000 RCF. Samples were dried to complete dryness and reconstituted in with MeOH/chloroform (100 μL, 9/1, v/v). For mass spectrometric analysis, sample (10 μL) was then added to MeOH/chloroform (90 uL) containing 7.5 mM ammonium acetate in a well of a 96 well plate and then sealed with aluminum foil. Mass spectrometric analysis was performed on a TSQ Quantum Ultra Plus triple-quadrupole mass spectrometer (Thermo Fisher Scientific) equipped with an automated nanospray apparatus (Nanomate HD, Advion Bioscience Ltd.). MS survey scans were acquired in both positive and negative ion mode. These scans were processed with GeneData Expressionist Refiner MS software (GeneData) and further validated using the NIST MS Search program to compare MS/MS data with an in-house library called LipidBlast^59^. Intensities were further analyzed using Statistica 9.0 software (StatSoft). Heatmap was created with the assistance of R 3.0.1 ( http://www.r-project.org/), using Euclidean distance measures and the Ward clustering algorithm.

### Cytotoxicity assay

HBE1 cells were seeded at 10,000 cells per well of 96-well plates and grown overnight. Cells were exposed to simvastatin, C18:1 lysophosphatidylcholine at indicated concentrations or vehicle control in growth medium for 24 hours. The exposed cells were then treated with pneumolysin at indicated concentrations for 4 hours and cell viability was analyzed with CellTiter-Glo® Luminescent Cell Viability Assay (Promega) following recommended protocol.

### Real-time RT-PCR

The procedures for RNA extraction and cDNA generation were described in our previous study^60^. Maxima SYBR Green qPCR Master Mix no Rox (Thermo Scientific) was used to perform Real-time PCR reactions on an Applied Biosystems 7900HT. Expressions were normalized to GAPDH for all reactions. Sequences of primers used in this study are included in Table ST3.

## Additional Information

**How to cite this article**: Statt, S. *et al.* Lipidome and Transcriptome Profiling of Pneumolysin Intoxication Identifies Networks Involved in Statin-Conferred Protection of Airway Epithelial Cells. *Sci. Rep.* **5**, 10624; doi: 10.1038/srep10624 (2015).

## Supplementary Material

Supporting Information

## Figures and Tables

**Table 1:** Statistically significant lipids from mass spectrometry data. Intensities were normalized by class. Statistically significant differences were assessed by one-way ANOVA with post hoc Dunnett’s correction and represented by asterisks (ns P > 0.05, ^*^P < 0.05, ^**^P < 0.01, ^***^P < 0.001).

**Lipid**	**p Value**	**Control vs Toxin**	**Control vs Statin**	**Control vs Both**	**Toxin vs Both**	**Statin vs Both**	**Statin vs Toxin**
plasmenyl-PC 38:4	0.000002	ns	***	***	*	ns	***
PC 30:0	0.000004	***	***	*	ns	**	ns
PC 32:1	0.000006	***	ns	ns	***	*	ns
PA 34:2	0.00001	***	***	ns	ns	**	ns
PC 32:0	0.000016	***	**	ns	***	**	ns
PC 34:2	0.000018	^***^	^***^	ns	^**^	^**^	ns
PC 36:1	0.000038	^***^	^*^	ns	^***^	^*^	ns
PC 36:2	0.000078	^***^	^*^	ns	^***^	^*^	ns
PS 40:2	0.000088	^*^	ns	ns	^*^	ns	^***^
PS 38:2	0.000093	ns	^*^	ns	^**^	ns	^***^
TG 52:4	0.000242	ns	^**^	^**^	^*^	^**^	^*^
PC 37:4	0.000566	ns	^**^	^**^	ns	ns	^*^
PC 35:4	0.000694	ns	^***^	^*^	ns	ns	^*^
lysoPC 18:1	0.001047	ns	ns	ns	^**^	ns	^**^
plasmenyl-PC 38:5	0.001055	^**^	^**^	ns	ns	ns	ns
TG 52:3	0.001548	ns	ns	ns	^**^	^**^	^**^
PC 36:2	0.001665	^***^	^*^	ns	^***^	^*^	ns
TG 54:4	0.001816	ns	^**^	^**^	ns	ns	ns
PG 36:1	0.003718	^***^	^*^	ns	^***^	^*^	ns
PS 36:1	0.004565	ns	ns	ns	ns	ns	^*^
PS 39:8	0.004883	^*^	ns	ns	ns	ns	^*^
PC 30:1	0.00537	ns	ns	^**^	^*^	ns	ns
PS 32:1	0.005472	ns	ns	^**^	ns	ns	ns
PG 34:1	0.007567	ns	ns	ns	ns	ns	^*^

**Table 2:** Top twenty differentially expressed genes from the mRNA-seq data, as compared to no treated controls. mRNA-seq reads were normalized using scaling factors for library size. P values were determined via a generalized linear model (GLM) likelihood ratio test within the edgeR package.

**Gene Symbol**	**Gene Definition**	**Log2 Fold Change, Treatment/None**	**p Value**
**TOXIN**			
EGR1	Early growth response 1	5.0821124356	1.02E-05
EGR2	Early growth response 2	4.5849534080	5.40E-06
IL20	Interleukin 20	4.5784428610	0.0002824629
C11orf96	Chromosome 11 open reading frame 96	4.4944040287	4.75E-06
ATF3	Activating transcription factor 3	4.4694850002	8.68E-06
TNF	Tumor necrosis factor	3.8800929090	0.0004175837
GEM	GTP binding protein overexpressed in skeletal muscle	3.4577584126	3.08E-05
NR4A1	Nuclear receptor subfamily 4, group A, member 1	3.2596075994	8.62E-07
CXCL2	Chemokine (C-X-C motif) ligand 2	3.2279840457	8.24E-05
IL8	Interleukin 8	3.2133483633	7.24E-06
NR4A3	Nuclear receptor subfamily 4, group A, member 3	3.0059522416	0.0078231198
FOSB	FBJ murine osteosarcoma viral oncogene homolog B	2.8638213316	0.0002123400
CREBRF	CREB3 regulatory factor	2.2232930679	9.67E-05
GADD45B	Growth arrest and DNA-damage-inducible, beta	2.2001574628	1.39E-08
GDF15	Growth differentiation factor 15	2.1755167968	6.21E-05
RND1	Rho family GTPase 1	2.0872777098	1.49E-08
PPP1R15A	Protein phosphatase 1, regulatory subunit 15A	2.0412893595	2.04E-05
BIRC3	Baculoviral IAP repeat containing 3	1.9263031522	0.0018212122
MAP2K6	Mitogen-activated protein kinase kinase 6	1.8155637257	0.0086922392
FOS	FBJ murine osteosarcoma viral oncogene homolog	1.7937408311	2.40E-07

**STATIN**			
FXYD3	FXYD domain containing ion transport regulator 3	4.433078947	1.45E-05
C11orf96	Chromosome 11 open reading frame 96	3.72461041	0.000145175
TNF	Tumor necrosis factor	3.695235714	0.001722366
NFASC	Neurofascin	3.635372308	8.42E-05
C6orf223	Chromosome 6 open reading frame 223	3.528111821	0.001664323
CD9	CD9 molecule	3.305106179	0.007018955
MAFF	V-maf avian musculoaponeurotic fibrosarcoma oncogene homolog F	3.147336747	3.14E-06
ATF3	Activating transcription factor 3	3.126219473	0.002610577
CXCL2	Chemokine (C-X-C motif) ligand 2	3.034548443	6.97E-06
SLC27A2	Solute carrier family 27 (fatty acid transporter), member 2	2.898860433	0.001683999
HSD17B7	Hydroxysteroid (17-beta) dehydrogenase 7	2.824996536	9.03E-06
REC8	REC8 meiotic recombination protein	2.793153813	1.23E-05
SLC29A2	Solute carrier family 29 (equilibrative nucleoside transporter), member 2	2.688913638	0.003246542
IL8	Interleukin 8	2.4637838	0.000248558
TM7SF2	Transmembrane 7 superfamily member 2, Delta(14)-sterol reductase	2.41216306	0.000137761
LGALS7B	Lectin, galactoside-binding, soluble, 7B	2.338793329	0.000115045
FDPS	Farnesyl diphosphate synthase	2.245113522	0.005511471
ACACB	Acetyl-CoA carboxylase beta	2.18279627	0.007533777
GPX2	Glutathione peroxidase 2	2.143697892	0.008187339
SSBP2	Single-stranded DNA binding protein 2	2.136914726	1.60E-05

**STATIN+TOXIN**			
EGR2	Early growth response 2	9.003110027	8.48E-23
C11orf96	Chromosome 11 open reading frame 96	8.51357552	1.45E-14
C6orf223	Chromosome 6 open reading frame 223	7.494553998	1.30E-10
ATF3	Activating transcription factor 3	7.208837244	8.96E-11
EGR1	Early growth response 1	7.163627625	7.66E-09
NR4A1	Nuclear receptor subfamily 4, group A, member 1	6.922380993	7.32E-20
IL8	Interleukin 8	6.778865409	5.51E-16
IL20	Interleukin 20	6.767729321	1.19E-07
TNF	Tumor necrosis factor	6.696143663	2.67E-08
FOSB	FBJ murine osteosarcoma viral oncogene homolog B	5.910606615	9.77E-12
CXCL2	Chemokine (C-X-C motif) ligand 2	5.589639741	3.35E-10
GEM	GTP binding protein overexpressed in skeletal muscle	5.479633585	4.96E-10
SOCS3	Suppressor of cytokine signaling 3	4.74582123	1.18E-17
ZNF460	Zinc finger protein 460	4.462020957	8.90E-12
PLA2G3	Phospholipase A2, group III	4.35384952	3.26E-06
TNFAIP3	Tumor necrosis factor, alpha-induced protein 3	4.253405429	3.07E-17
BIRC3	Baculoviral IAP repeat containing , apoptosis inhibitor	4.24819277	6.31E-10
KLF2	Kruppel-like factor 2	4.160537722	1.09E-08
RASGEF1B	RasGEF domain family, member 1B	4.15890662	8.45E-17
CCL20	Chemokine (C-C motif) ligand 20	4.067584886	1.09E-09

**Table 3:** Enriched Gene Ontology (GO) pathways in the transcriptome of treatment groups as compared to no treated controls. Nominal p values are calculated with the GSEA program by using an empirical phenotype-based permutation test. False discovery rate is adjusted for using an adapted Benjamini–Hochberg procedure.

**GO Canonical Pathway**	**Number of Genes in Set**	**NOM p-val**	**FDR q-val**
**TOXIN**			
RESPONSE TO EXTRACELLULAR STIMULUS	22	0	0.002938457
NITROGEN COMPOUND CATABOLIC PROCESS	18	0	0.007242801
CATION TRANSPORT	61	0	0.019453308
RESPONSE TO NUTRIENT LEVELS	18	0	0.019539576
INFLAMMATORY RESPONSE	65	0	0.020307241
TRANSLATION	151	0	0.024203578
STEROID METABOLIC PROCESS	43	0	0.02606539
MONOCARBOXYLIC ACID METABOLIC PROCESS	65	0	0.039081186
GLYCEROPHOSPHOLIPID BIOSYNTHETIC PROCESS	29	0	0.04601446
LIPID METABOLIC PROCESS	227	0	0.04833658

**STATIN**			
MONOCARBOXYLIC ACID METABOLIC PROCESS	65	0	0
LIPID METABOLIC PROCESS	227	0	0.0018675
GLUCOSE METABOLIC PROCESS	21	0	0.002207291
FATTY ACID METABOLIC PROCESS	50	0	0.002241
CELLULAR LIPID METABOLIC PROCESS	185	0	0.002287963
STEROID METABOLIC PROCESS	43	0	0.003534764
FATTY ACID OXIDATION	16	0	0.006060679
CARBOXYLIC ACID METABOLIC PROCESS	135	0	0.006493585
LIPID BIOSYNTHETIC PROCESS	80	0	0.006537986
INFLAMMATORY RESPONSE	65	0	0.03650236

**STATIN+TOXIN**			
LIPID METABOLIC PROCESS	227	0	0.007665763
CATION TRANSPORT	61	0	0.021874871
GLUCOSE METABOLIC PROCESS	21	0	0.023219533
CELLULAR LIPID METABOLIC PROCESS	185	0	0.030281333
RESPONSE TO EXTERNAL STIMULUS	164	0	0.032070216
MONOCARBOXYLIC ACID METABOLIC PROCESS	65	0	0.03301661
RESPONSE TO NUTRIENT LEVELS	18	0.008547009	0.04540056
DEFENSE RESPONSE	126	0	0.04604433
MEMBRANE LIPID BIOSYNTHETIC PROCESS	44	0.007407407	0.047668364
INFLAMMATORY RESPONSE	65	0	0.048929326

**Table 4:** Enriched Kyoto Encyclopedia of Genes and Genomes (KEGG) pathways in the transcriptome of treatment groups as compared to no treated controls. Nominal p values are calculated with the GSEA program by using an empirical phenotype-based permutation test. False discovery rate is adjusted for using an adapted Benjamini–Hochberg procedure.

**KEGG Canonical Pathway**	**Number of Genes in Set**	**NOM p-val**	**FDR q-val**
**TOXIN**			
RIBOSOME	85	0	0
GLUTATHIONE METABOLISM	41	0	0
LYSOSOME	110	0	0
BIOSYNTHESIS OF UNSATURATED FATTY ACIDS	18	0	0.008914938
STEROID BIOSYNTHESIS	15	0	0.013855341
LEISHMANIA INFECTION	43	0	0.014490775
ARACHIDONIC ACID METABOLISM	34	0	0.015463158
CARDIAC MUSCLE CONTRACTION	38	0	0.015587258
STEROID HORMONE BIOSYNTHESIS	30	0	0.017814009
METABOLISM OF XENOBIOTICS BY CYTOCHROME P450	42	0.00990099	0.021507936

**STATIN**			
STEROID BIOSYNTHESIS	15	0	0
BIOSYNTHESIS OF UNSATURATED FATTY ACIDS	18	0	0
ECM RECEPTOR INTERACTION	50	0	0
METABOLISM OF XENOBIOTICS BY CYTOCHROME P450	42	0	0
ADIPOCYTOKINE SIGNALING PATHWAY	52	0	0.001815
GLUTATHIONE METABOLISM	41	0	0.002716081
DRUG METABOLISM CYTOCHROME P450	39	0	0.005934439
STEROID HORMONE BIOSYNTHESIS	30	0	0.006539325
DILATED CARDIOMYOPATHY	44	0	0.007513437
PYRUVATE METABOLISM	31	0	0.008348262

**STATIN+TOXIN**			
RIBOSOME	85	0	0
STEROID BIOSYNTHESIS	15	0	0
BIOSYNTHESIS OF UNSATURATED FATTY ACIDS	18	0	0
METABOLISM OF XENOBIOTICS BY CYTOCHROME P450	42	0	0
GLUTATHIONE METABOLISM	41	0	0
STEROID HORMONE BIOSYNTHESIS	30	0	0
LYSOSOME	110	0	0.000965857
ADIPOCYTOKINE SIGNALING PATHWAY	52	0	0.009771074
PPAR SIGNALING PATHWAY	40	0	0.011506875
ARACHIDONIC ACID METABOLISM	34	0	0.011509539

## References

[b1] Rock J. R. & Hogan B. L. Epithelial progenitor cells in lung development, maintenance, repair, and disease. *Annual review of cell and developmental biology* 27, 493–512, 10.1146/annurev-cellbio-100109-104040 (2011). 21639799

[b2] Holt P. G., Strickland D. H. & Sly P. D. Virus infection and allergy in the development of asthma: what is the connection? Current opinion in allergy and clinical immunology 12, 151–157, 10.1097/ACI.0b013e3283520166 (2012). 22356945

[b3] Malley R. Antibody and cell-mediated immunity to Streptococcus pneumoniae: implications for vaccine development. J Mol Med (Berl) 88, 135–142, 10.1007/s00109-009-0579-4 (2010). 20049411

[b4] Chiavolini D., Pozzi G. & Ricci S. Animal models of Streptococcus pneumoniae disease. Clinical microbiology reviews 21, 666–685, 10.1128/CMR.00012-08 (2008). 18854486 PMC2570153

[b5] Lanata C. & Black R. in Nutrition and Health in Developing Countries Nutrition and Health Series (eds RichardD Semba, MartinW Bloem, & Peter Piot ) Ch. 7, 179–214 (Humana Press, 2008).

[b6] Johnson H. L. *et al.* Systematic evaluation of serotypes causing invasive pneumococcal disease among children under five: the pneumococcal global serotype project. PLoS Med 7, 10.1371/journal.pmed.1000348 (2010). PMC2950132 20957191

[b7] Tweten R. K. Cholesterol-dependent cytolysins, a family of versatile pore-forming toxins. Infection and immunity 73, 6199–6209, 10.1128/IAI.73.10.6199-6209.2005 (2005). 16177291 PMC1230961

[b8] Tilley S. J., Orlova E. V., Gilbert R. J., Andrew P. W. & Saibil H. R. Structural basis of pore formation by the bacterial toxin pneumolysin. Cell 121, 247–256, 10.1016/j.cell.2005.02.033 (2005). 15851031

[b9] Ramachandran R., Heuck A. P., Tweten R. K. & Johnson A. E. Structural insights into the membrane-anchoring mechanism of a cholesterol-dependent cytolysin. Nature structural biology 9, 823–827, 10.1038/nsb855 (2002). 12368903

[b10] Gonzalez M. R. *et al.* Pore-forming toxins induce multiple cellular responses promoting survival. Cellular microbiology 13, 1026–1043, 10.1111/j.1462-5822.2011.01600.x (2011). 21518219

[b11] Goldstein J. L. & Brown M. S. Regulation of the mevalonate pathway. Nature 343, 425–430, 10.1038/343425a0 (1990). 1967820

[b12] Majumdar S. R., McAlister F. A., Eurich D. T., Padwal R. S. & Marrie T. J. Statins and outcomes in patients admitted to hospital with community acquired pneumonia: population based prospective cohort study. BMJ 333, 999, 10.1136/bmj.38992.565972.7C (2006). 17060337 PMC1635620

[b13] Magulick J. P. *et al.* The Effect of Statin Therapy on the Incidence of Infections: A Retrospective Cohort Analysis. The American journal of the medical sciences , 10.1097/MAJ.0b013e31828318e2 (2013). PMC3664245 23426088

[b14] Pruefer D. *et al.* Simvastatin inhibits inflammatory properties of Staphylococcus aureus alpha-toxin. Circulation 106, 2104–2110 (2002). 12379581 10.1161/01.cir.0000034048.38910.91

[b15] Burns E. M. *et al.* Short Term Statin Treatment Improves Survival and Differentially Regulates Macrophage-Mediated Responses to Staphylococcus aureus. Current pharmaceutical biotechnology 14, 233–241 (2013). 23228241 10.2174/138920113805219395 PMC4467892

[b16] Chow O. A. *et al.* Statins enhance formation of phagocyte extracellular traps. Cell host & microbe 8, 445–454, 10.1016/j.chom.2010.10.005 (2010). 21075355 PMC3008410

[b17] Laaksonen R. *et al.* A systems biology strategy reveals biological pathways and plasma biomarker candidates for potentially toxic statin-induced changes in muscle. PLoS One 1, e97, 10.1371/journal.pone.0000097 (2006). 17183729 PMC1762369

[b18] Kaddurah-Daouk R. *et al.* Lipidomic analysis of variation in response to simvastatin in the Cholesterol and Pharmacogenetics Study. Metabolomics 6, 191–201, 10.1007/s11306-010-0207-x (2010). 20445760 PMC2862962

[b19] Chen F. *et al.* The effects of simvastatin treatment on plasma lipid-related biomarkers in men with dyslipidaemia. Biomarkers : biochemical indicators of exposure, response, and susceptibility to chemicals 16, 321–333, 10.3109/1354750X.2011.561367 (2011). 21417623

[b20] Fratta Pasini A. *et al.* Lysophosphatidylcholine and carotid intima-media thickness in young smokers: a role for oxidized LDL-induced expression of PBMC lipoprotein-associated phospholipase A2? PloS one 8, e83092, 10.1371/journal.pone.0083092 (2013). 24358251 PMC3866188

[b21] Audas T. E., Li Y., Liang G. & Lu R. A novel protein, Luman/CREB3 recruitment factor, inhibits Luman activation of the unfolded protein response. Molecular and cellular biology 28, 3952–3966, 10.1128/MCB.01439-07 (2008). 18391022 PMC2423117

[b22] Myles I. A. *et al.* Signaling via the IL-20 receptor inhibits cutaneous production of IL-1beta and IL-17A to promote infection with methicillin-resistant Staphylococcus aureus. Nature immunology 14, 804–811, 10.1038/ni.2637 (2013). 23793061 PMC3721434

[b23] Murakami M. & Lambeau G. Emerging roles of secreted phospholipase A(2) enzymes: an update. Biochimie 95, 43–50, 10.1016/j.biochi.2012.09.007 (2013). 23022039

[b24] Osborne T. F. Sterol regulatory element-binding proteins (SREBPs): key regulators of nutritional homeostasis and insulin action. The Journal of biological chemistry 275, 32379–32382, 10.1074/jbc.R000017200 (2000). 10934219

[b25] Janky R. *et al.* iRegulon: From a Gene List to a Gene Regulatory Network Using Large Motif and Track Collections. PLoS computational biology 10, e1003731, 10.1371/journal.pcbi.1003731 (2014). 25058159 PMC4109854

[b26] Subramanian A. *et al.* Gene set enrichment analysis: a knowledge-based approach for interpreting genome-wide expression profiles. Proceedings of the National Academy of Sciences of the United States of America 102, 15545–15550, 10.1073/pnas.0506580102 (2005). 16199517 PMC1239896

[b27] van der Poll T. *et al.* Interleukin-6 gene-deficient mice show impaired defense against pneumococcal pneumonia. The Journal of infectious diseases 176, 439–444 (1997). 9237710 10.1086/514062

[b28] Endo A. The discovery and development of HMG-CoA reductase inhibitors. Journal of lipid research 33, 1569–1582 (1992). 1464741

[b29] Chen J. C., Wu M. L., Huang K. C. & Lin W. W. HMG-CoA reductase inhibitors activate the unfolded protein response and induce cytoprotective GRP78 expression. Cardiovascular research 80, 138–150, 10.1093/cvr/cvn160 (2008). 18556704

[b30] Wajant H., Pfizenmaier K. & Scheurich P. Tumor necrosis factor signaling. Cell death and differentiation 10, 45–65, 10.1038/sj.cdd.4401189 (2003). 12655295

[b31] Dragneva Y. *et al.* Subcytocidal attack by staphylococcal alpha-toxin activates NF-kappaB and induces interleukin-8 production. Infection and immunity 69, 2630–2635, 10.1128/IAI.69.4.2630-2635.2001 (2001). 11254628 PMC98200

[b32] Chopra A. K. *et al.* The cytotoxic enterotoxin of Aeromonas hydrophila induces proinflammatory cytokine production and activates arachidonic acid metabolism in macrophages. Infection and immunity 68, 2808–2818 (2000). 10768977 10.1128/iai.68.5.2808-2818.2000 PMC97492

[b33] Fickl H. *et al.* Pneumolysin-mediated activation of NFkappaB in human neutrophils is antagonized by docosahexaenoic acid. Clinical and experimental immunology 140, 274–281, 10.1111/j.1365-2249.2005.02757.x (2005). 15807851 PMC1809376

[b34] Kayal S. *et al.* Listeriolysin O secreted by Listeria monocytogenes induces NF-kappaB signalling by activating the IkappaB kinase complex. Molecular microbiology 44, 1407–1419 (2002). 12028384 10.1046/j.1365-2958.2002.02973.x

[b35] Matsumoto M., Einhaus D., Gold E. S. & Aderem A. Simvastatin augments lipopolysaccharide-induced proinflammatory responses in macrophages by differential regulation of the c-Fos and c-Jun transcription factors. J Immunol 172, 7377–7384 (2004). 15187114 10.4049/jimmunol.172.12.7377

[b36] Montero M. T. *et al.* Hydroxymethylglutaryl-coenzyme A reductase inhibition stimulates caspase-1 activity and Th1-cytokine release in peripheral blood mononuclear cells. Atherosclerosis 153, 303–313 (2000). 11164419 10.1016/s0021-9150(00)00417-2

[b37] Dennis E. A., Cao J., Hsu Y. H., Magrioti V. & Kokotos G. Phospholipase A2 enzymes: physical structure, biological function, disease implication, chemical inhibition, and therapeutic intervention. Chemical reviews 111, 6130–6185, 10.1021/cr200085w (2011). 21910409 PMC3196595

[b38] Triggiani M., Granata F., Frattini A. & Marone G. Activation of human inflammatory cells by secreted phospholipases A2. Biochimica et biophysica acta 1761, 1289–1300, 10.1016/j.bbalip.2006.07.003 (2006). 16952481

[b39] Henderson W. R. Jr. *et al.* Blockade of human group X secreted phospholipase A2 (GX-sPLA2)-induced airway inflammation and hyperresponsiveness in a mouse asthma model by a selective GX-sPLA2 inhibitor. The Journal of biological chemistry 286, 28049–28055, 10.1074/jbc.M111.235812 (2011). 21652694 PMC3151050

[b40] Foreman-Wykert A. K., Weinrauch Y., Elsbach P. & Weiss J. Cell-wall determinants of the bactericidal action of group IIA phospholipase A2 against Gram-positive bacteria. The Journal of clinical investigation 103, 715–721, 10.1172/JCI5468 (1999). 10074489 PMC408128

[b41] Koduri R. S. *et al.* Bactericidal properties of human and murine groups I, II, V, X, and XII secreted phospholipases A(2). The Journal of biological chemistry 277, 5849–5857, 10.1074/jbc.M109699200 (2002). 11694541

[b42] Menschikowski M. *et al.* Statins potentiate the IFN-gamma-induced upregulation of group IIA phospholipase A2 in human aortic smooth muscle cells and HepG2 hepatoma cells. Biochimica et biophysica acta 1733, 157–171, 10.1016/j.bbalip.2005.01.001 (2005). 15863363

[b43] Laine V. J., Grass D. S. & Nevalainen T. J. Protection by group II phospholipase A2 against Staphylococcus aureus. J Immunol 162, 7402–7408 (1999). 10358193

[b44] Harwig S. S. *et al.* Bactericidal properties of murine intestinal phospholipase A2. The Journal of clinical investigation 95, 603–610, 10.1172/JCI117704 (1995). 7860744 PMC295524

[b45] Qu X. D. & Lehrer R. I. Secretory phospholipase A2 is the principal bactericide for staphylococci and other gram-positive bacteria in human tears. Infection and immunity 66, 2791–2797 (1998). 9596749 10.1128/iai.66.6.2791-2797.1998 PMC108271

[b46] Statt S. *et al.* Statins Enhance Cellular Resistance Against Bacterial Pore-forming Toxins in Airway Epithelial Cells. American journal of respiratory cell and molecular biology , 10.1165/rcmb.2014-0391OC (2015). PMC4742951 25874372

[b47] Dennis E. A. *et al.* A mouse macrophage lipidome. The Journal of biological chemistry 285, 39976–39985, 10.1074/jbc.M110.182915 (2010). 20923771 PMC3000979

[b48] Coles S. J., Bhaskar K. R., O’Sullivan D. D., Neill K. H. & Reid L. M. Airway mucus: composition and regulation of its secretion by neuropeptides *in vitro*. Ciba Foundation symposium 109, 40–60 (1984). 6083850 10.1002/9780470720905.ch4

[b49] Periole X., Huber T., Marrink S. J. & Sakmar T. P. G protein-coupled receptors self-assemble in dynamics simulations of model bilayers. Journal of the American Chemical Society 129, 10126–10132, 10.1021/ja0706246 (2007). 17658882

[b50] Crimi M. & Esposti M. D. Apoptosis-induced changes in mitochondrial lipids. Biochimica et biophysica acta 1813, 551–557, 10.1016/j.bbamcr.2010.09.014 (2011). 20888373

[b51] Degli Esposti M. Sequence and functional similarities between pro-apoptotic Bid and plant lipid transfer proteins. Biochimica et biophysica acta 1553, 331–340 (2002). 11997142 10.1016/s0005-2728(02)00187-1

[b52] Kuwana T. *et al.* Bid, Bax, and lipids cooperate to form supramolecular openings in the outer mitochondrial membrane. Cell 111, 331–342 (2002). 12419244 10.1016/s0092-8674(02)01036-x

[b53] Stormo C. *et al.* RNA-sequencing analysis of HepG2 cells treated with atorvastatin. *PloS one* **9** , e105836, 10.1371/journal.pone.0105836 (2014). PMC4143339 25153832

[b54] Ha U. H. *et al.* MKP1 regulates the induction of MUC5AC mucin by Streptococcus pneumoniae pneumolysin by inhibiting the PAK4-JNK signaling pathway. The Journal of biological chemistry 283, 30624–30631, 10.1074/jbc.M802519200 (2008). 18782768 PMC2576563

[b55] Li H. & Durbin R. Fast and accurate long-read alignment with Burrows-Wheeler transform. Bioinformatics 26, 589–595, 10.1093/bioinformatics/btp698 (2010). 20080505 PMC2828108

[b56] Robinson M. D., McCarthy D. J. & Smyth G. K. edgeR: a Bioconductor package for differential expression analysis of digital gene expression data. Bioinformatics 26, 139–140, 10.1093/bioinformatics/btp616 (2010). 19910308 PMC2796818

[b57] Shannon P. *et al.* Cytoscape: a software environment for integrated models of biomolecular interaction networks. Genome research 13, 2498–2504, 10.1101/gr.1239303 (2003). 14597658 PMC403769

[b58] Matyash V., Liebisch G., Kurzchalia T. V., Shevchenko A. & Schwudke D. Lipid extraction by methyl-tert-butyl ether for high-throughput lipidomics. Journal of lipid research 49, 1137–1146, 10.1194/jlr.D700041-JLR200 (2008). 18281723 PMC2311442

[b59] Kind T. *et al.* LipidBlast in silico tandem mass spectrometry database for lipid identification. Nature methods 10, 755–758, 10.1038/nmeth.2551 (2013). 23817071 PMC3731409

[b60] Kao C. Y. *et al.* IL-17 markedly up-regulates beta-defensin-2 expression in human airway epithelium via JAK and NF-kappaB signaling pathways. J Immunol 173, 3482–3491 (2004). 15322213 10.4049/jimmunol.173.5.3482

