# Supplementary material for: Lipidome and Transcriptome Profiling of Pneumolysin Intoxication Identifies Networks Involved in Statin-Conferred Protection of Airway Epithelial Cells
Source: Sci Rep. 2015 May 29;5:10624. doi: 10.1038/srep10624 (PMC4448502; doi:10.1038/srep10624)
Supplement: Supporting Information [file srep10624-s1.pdf]

Lipidome and Transcriptome Profiling of Pneumolysin Intoxication Identifies Networks Involved in Statin-Conferred Protection of Airway Epithelial Cells

Sarah Statt[1], Jhen-Wei Ruan[2], Chih-Ting Huang[2], Reen Wu[1], and Cheng-Yuan Kao[1,2] *

Affiliations

[1] Center for Comparative Respiratory Biology and Medicine, University of California at Davis

Davis, California 95616

[2] Immunology Research Center, National Health Research Institutes, Zhunan, Miaoli 35053, Taiwan

*Correspondence to: Cheng-Yuan Kao, Ph.D., Current affiliation: Immunology Research Center, National Health Research Institutes, Zhunan, Miaoli 35053, Taiwan, Tel: +886-37-246166 ext 37622; Fax: +886-37-586642; E- mail: chengyuankao@nhri.org.tw; previous affiliation: Center for Comparative Respiratory Biology and Medicine, University of California, Davis, Davis, CA 95616, Tel: 1-530-754-6932; Fax: 1-530-752-8632

Running title: Profiling Airway Statin-Pneumolysin Interaction

Keywords: Pneumolysin, Statin, Lipidomics, RNA-seq, Airway Epithelium

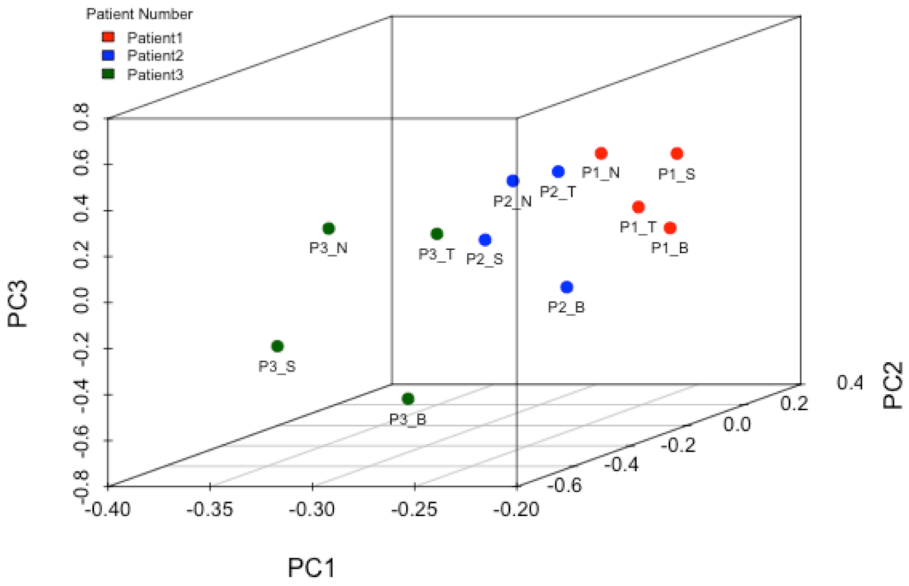

Figure S1: Three-dimensional principal component analysis of mRNA-seq data showed biological variation stronger than treatment. Lung epithelial cells from three patients with no known lung diseases underwent the following treatments: no treatment/vehicle control, pneumolysin (400 ng/mL) alone for 4 hours, simvastatin (1μM) 24 hour treatment alone and both simvastatin (1μM) 24 hour pretreatment and pneumolysin (400 ng/mL) for 4 hours. The x-axis represents the eigenvalue (EV) for principal component 1 (PC1), the y-axis represents the eigenvalue for principal component 3 (PC3) and the z-axis represents the eigenvalue for principal component 2 (PC2). Red represents all samples from Patient 1, blue represents all samples from Patient 2 and green represents all samples from Patient 3.

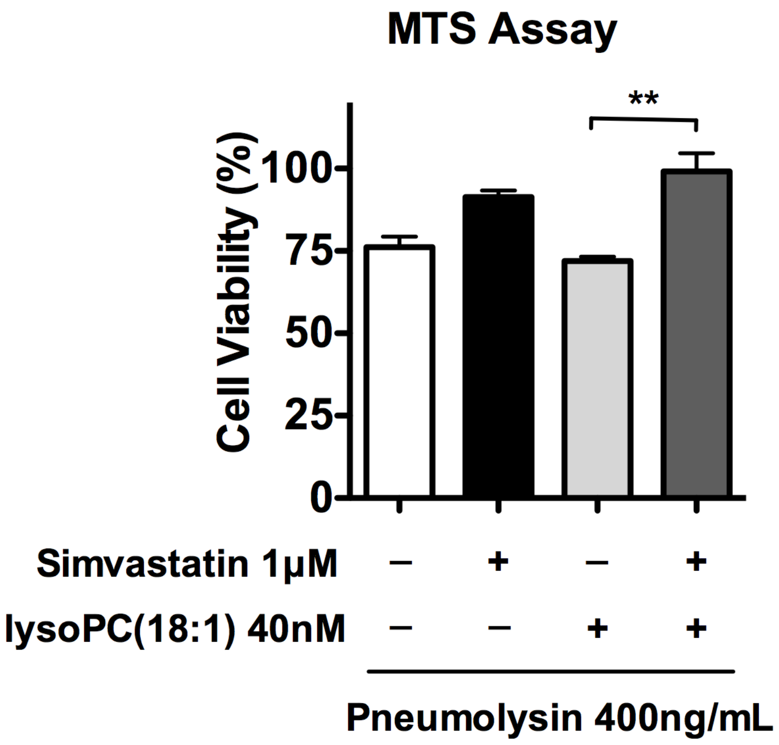

Figure S2: LysoPC 18:1 did not abrogate simvastatin-mediated cellular protection against pneumolysin. HBE1 cells were pretreated with 1μM simvastatin with or without 40nM of lysoPC 18:1 for 24 hours and then challenged with 400ng/mL pneumolysin for 4 hours. The MTS assay was then performed to determine cell viability. Asterisks indicate significant statistical difference versus matching groups with one-way ANOVA and Tukey's post hoc test. Error bars represent S.E.M. of 3 experiments.

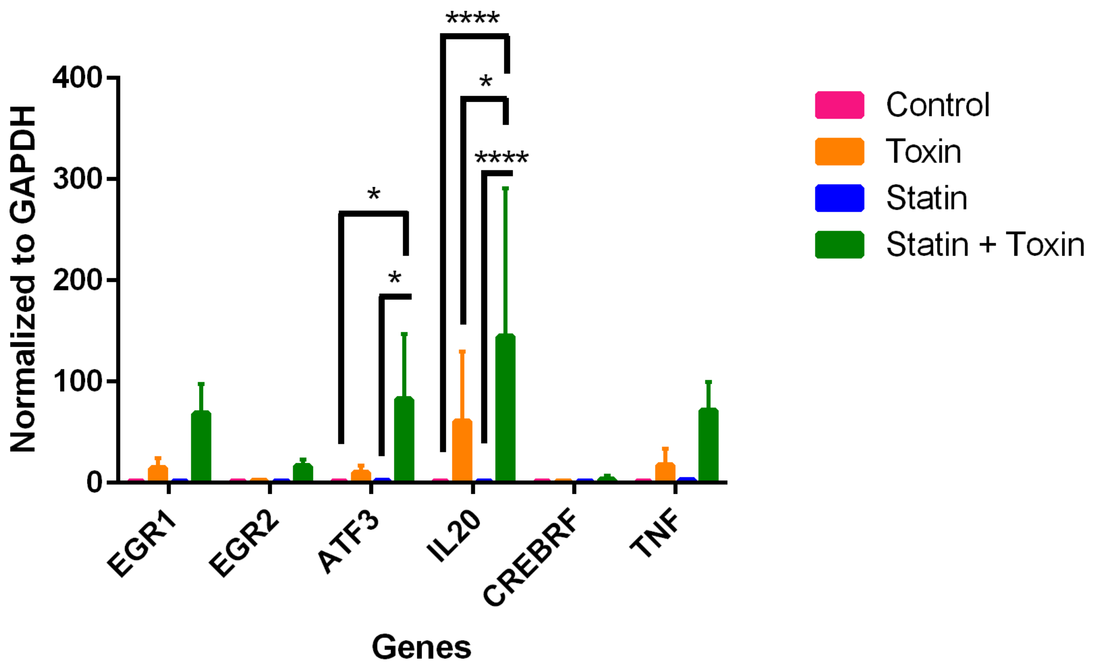

Figure S3: RT-qPCR validation of differential expression genes. Corresponding average mRNA expression for EGR1, EGR2, ATF3, IL20, CREBRF and TNF, using GAPDH as internal control is shown. Error bars represent standard deviation of three replicates. Asterisks indicate significant statistical difference versus matching groups with one-way ANOVA and Tukey's post hoc test.

Table ST1. 132 Unique Lipids and 22 Metabolites Found in Lipidomics Analysis

**Cardiolipins (CL)**	**Phosphatidylcholines (PC)**
Cardiolipin C60:1	Lysophosphatidylcholine C9:0
Cardiolipin C66:3	Lysophosphatidylcholine C11:0
Cardiolipin C68:2	Lysophosphatidylcholine C17:1
Cardiolipin C70:2	Lysophosphatidylcholine C18:0
Cardiolipin C72:5	Lysophosphatidylcholine C18:1
Cardiolipin C74:2	Plasmenyl-Phosphatidylcholine C29:0
Cardiolipin C76:9	Plasmenyl-Phosphatidylcholine C30:0
Cardiolipin C78:8	Plasmenyl-Phosphatidylcholine C31:0
Cardiolipin C78:14	Plasmenyl-Phosphatidylcholine C31:1
Cardiolipin C80:11	Plasmenyl-Phosphatidylcholine C32:0
Cardiolipin C80:13	Plasmenyl-Phosphatidylcholine C32:1
**Diacylglycerophosphates (PA)**	Plasmenyl-Phosphatidylcholine C33:0
Diacylglycerophosphate C8:0	Plasmenyl-Phosphatidylcholine C34:0
Diacylglycerophosphate C12:0	Plasmenyl-Phosphatidylcholine C34:2
Diacylglycerophosphate C34:2	Plasmenyl-Phosphatidylcholine C35:2
**Phosphatidylethanolamines (PE)**	Plasmenyl-Phosphatidylcholine C36:0
Plasmenyl-Phosphatidylethanolamine C32:1	Plasmenyl-Phosphatidylcholine C36:1
Plasmenyl-Phosphatidylethanolamine C34:2	Plasmenyl-Phosphatidylcholine C37:0
Phosphatidylethanolamine C12:0	Plasmenyl-Phosphatidylcholine C38:0
Phosphatidylethanolamine C21:0	Plasmenyl-Phosphatidylcholine C38:1
Phosphatidylethanolamine C32:1	Plasmenyl-Phosphatidylcholine C38:2
Phosphatidylethanolamine C34:1	Plasmenyl-Phosphatidylcholine C38:4
Phosphatidylethanolamine C34:2	Plasmenyl-Phosphatidylcholine C38:5
Phosphatidylethanolamine C36:1	Phosphatidylcholine C7:0
Phosphatidylethanolamine C36:2	Phosphatidylcholine C10:0
Phosphatidylethanolamine C38:2	Phosphatidylcholine C28:0
Phosphatidylethanolamine C38:3	Phosphatidylcholine C29:0
Phosphatidylethanolamine C38:4	Phosphatidylcholine C30:0
**Phosphatidylglycerols (PG)**	Phosphatidylcholine C30:1
Phosphatidylglycerol C34:1	Phosphatidylcholine C30:2
Phosphatidylglycerol C34:2	Phosphatidylcholine C31:0
Phosphatidylglycerol C36:1	Phosphatidylcholine C32:0
Phosphatidylglycerol C36:2	Phosphatidylcholine C32:1
Phosphatidylglycerol C38:2	Phosphatidylcholine C32:2
**Phosphatidylinositols (PI)**	Phosphatidylcholine C33:1
Phosphatidylinositol C31:0	Phosphatidylcholine C33:2
Phosphatidylinositol C31:1	Phosphatidylcholine C34:1
Phosphatidylinositol C32:1	Phosphatidylcholine C34:2
Phosphatidylinositol C33:1	Phosphatidylcholine C34:3
Phosphatidylinositol C33:2	Phosphatidylcholine C35:0
Phosphatidylinositol C34:0	Phosphatidylcholine C35:3
Phosphatidylinositol C34:1	Phosphatidylcholine C35:4
Phosphatidylinositol C34:2	Phosphatidylcholine C36:1
Phosphatidylinositol C35:1	Phosphatidylcholine C36:2
Phosphatidylinositol C35:2	Phosphatidylcholine C37:1
Phosphatidylinositol C36:1	Phosphatidylcholine C37:2
Phosphatidylinositol C36:2	Phosphatidylcholine C37:3
Phosphatidylinositol C36:3	Phosphatidylcholine C37:4
Phosphatidylinositol C37:4	Phosphatidylcholine C37:5
Phosphatidylinositol C38:2	Phosphatidylcholine C38:2
Phosphatidylinositol C38:3	Phosphatidylcholine C38:3
Phosphatidylinositol C38:4	Phosphatidylcholine C40:10

**Phosphatidylserines (PS)**	**Triacylglycerols (TG)**
Phosphatidylserine C32:0	Triacylglycerol C50:2
Phosphatidylserine C32:1	Triacylglycerol C52:3
Phosphatidylserine C34:0	Triacylglycerol C52:4
Phosphatidylserine C34:1	Triacylglycerol C54:2
Phosphatidylserine C34:2	Triacylglycerol C54:4
Phosphatidylserine C36:0	**Metabolites**
Phosphatidylserine C36:1	alpha-D-Glucose 1,6-bisphosphate
Phosphatidylserine C36:2	1-Hexadecanoyl-2-octadecenoyl-sn-glycero-3-phosphocholine
Phosphatidylserine C38:2	1,2-Dipalmitoyl-sn-glycero-3-phosphocholine
Phosphatidylserine C39:8	17.alpha.-Hydroxypregnenolone
Phosphatidylserine C40:2	2-Propen-1-one, 1-[2,4-dihydroxy-6-methoxy-3-(3-methyl-2-buten-1-yl)phenyl]-3-(4-hydroxyphenyl)-, (2E)-
**Sphingomyelins (SM)**	8-(2-Aminoethylthio)guanosine-3',5'-cyclic monophosphate
Sphingomyelin C34:0	Azoxystrobin
Sphingomyelin C34:1	Benzyl hexadecyl dimethylammonium cation
Sphingomyelin C34:2	Bis(2-ethylhexyl) phthalate
Sphingomyelin C35:0	Cevane-3,4,7,14,15,16,20-heptol, 4,9-epoxy-, 7-acetate 3,15-bis[(2Z)-2-methyl-2-butenoate], (3.beta.,4.alpha.,7.alpha.,15.alpha.,16.beta.)-
Sphingomyelin C36:1	Decamethylcyclopentasiloxane
Sphingomyelin C37:1	Diafenthiuron
Sphingomyelin C38:0	Dihydrorobustic Acid
Sphingomyelin C39:1	Fluazifop-p-butyl
Sphingomyelin C42:2	Griseofulvin
N-Stearoyl-4-sphingenyl-1-O-phosphorylcholine	His-Thr-Lys
N-Tetracosanoyl-4-sphingenyl-1-O-phosphorylcholine	Nabilone
**Eicosanoids**	Quercetin Tetramethyl (5,7,3',4') Ether
Prostaglandin H2	Scopoletin
**Sterol Lipids**	Terazosin
Cholecalciferol	Trimethylsilyl temazepam
Cholesterol	Val-Met-Arg
7-ketocholesterol	
3-Oxocholestane	

Table ST2. Statistics for the 93 Lipid Intensities

Name	Control Average Normalized Intensity (+/- SD)	Toxin Average Normalized Intensity (+/- SD)	Statin Average Normalized Intensity (+/- SD)	Statin + Toxin Average Normalized Intensity (+/- SD)	Control vs. Toxin		Control vs. Statin		Control vs. Statin + Toxin	
					p value	Fold Change	p value	Fold Change	p value	Fold Change
lysoPC 9:0	704 +/- 264	863 +/- 294	785 +/- 365	960 +/- 381	0.8051	1.23	0.9668	1.12	0.4809	1.36
lysoPC 11:0	846 +/- 333	1320 +/- 304	1140 +/- 236	1543 +/- 893	0.3323	1.56	0.7100	1.35	0.0794	1.82
lysoPC 18:0	1472 +/- 504	1862 +/- 927	1057 +/- 398	1658 +/- 1156	0.8038	1.26	0.7718	0.72	0.9726	1.13
lysoPC 18:1	9942 +/- 3901	13633 +/- 4362	7112 +/- 1989	6272 +/- 1753	0.1665	1.37	0.3720	0.72	0.1701	0.63
PA 12:0	125 +/- 45	193 +/- 72	137 +/- 30	137 +/- 41	0.0760	1.54	0.9669	1.10	0.9727	1.09
PA 34:2	17283 +/- 1352	22686 +/- 1431	23832 +/- 2474	19732 +/- 2488	0.0004	1.31	0.0002	1.38	0.1317	1.14
PC 29:0	10370 +/- 2407	10687 +/- 1100	11415 +/- 1396	9629 +/- 1597	0.9849	1.03	0.6613	1.10	0.8459	0.93
PC 30:0	64123 +/- 10206	96277 +/- 8162	110706 +/- 15481	84299 +/- 15049	0.0005	1.50	0.0002	1.73	0.0302	1.31
PC 30:1	20298 +/- 6039	18584 +/- 2213	16709 +/- 727	13298 +/- 2107	0.7827	0.92	0.2261	0.82	0.0041	0.66
PC 30:2	6270 +/- 1640	6513 +/- 706	6331 +/- 875	6002 +/- 946	0.9758	1.04	0.9996	1.01	0.9679	0.96
PC 31:0	24080 +/- 4074	26545 +/- 2116	29691 +/- 3031	24421 +/- 3293	0.4885	1.10	0.0159	1.23	0.9972	1.01
PC 32:0	38842 +/- 5592	53099 +/- 5745	49300 +/- 4311	38578 +/- 4934	0.0003	1.37	0.0049	1.27	0.9997	0.99
PC 32:0	27342 +/- 3731	33450 +/- 3916	45582 +/- 7487	35218 +/- 6217	0.1975	1.22	0.0002	1.67	0.0634	1.29
PC 32:1	161160 +/- 25238	235818 +/- 26075	197604 +/- 16414	153371 +/- 30402	0.0002	1.46	0.0542	1.23	0.9367	0.95
PC 32:2	16925 +/- 4029	19721 +/- 4561	19486 +/- 2405	16843 +/- 3319	0.4962	1.17	0.5682	1.15	1.0000	1.00
PC 33:2	25003 +/- 6393	31404 +/- 8229	23368 +/- 2975	22950 +/- 5836	0.2365	1.26	0.9590	0.93	0.9233	0.92
PC 34:1	35665 +/- 12504	33360 +/- 3507	35470 +/- 2748	27996 +/- 6486	0.9361	0.94	1.0000	0.99	0.2377	0.78
PC 34:2	155118 +/- 24299	228089 +/- 22041	223465 +/- 21766	171953 +/- 33731	0.0003	1.47	0.0004	1.44	0.6237	1.11
PC 34:3	6313 +/- 1466	7593 +/- 2157	8658 +/- 1392	8298 +/- 845	0.4210	1.20	0.0409	1.37	0.1012	1.31
PC 35:0	6473 +/- 1454	7323 +/- 779	7285 +/- 758	7409 +/- 1463	0.5329	1.13	0.5696	1.13	0.4516	1.14
PC 35:3	15778 +/- 5063	21455 +/- 5198	23670 +/- 7261	21750 +/- 6006	0.3043	1.36	0.0885	1.50	0.2634	1.38
PC 35:4	9110 +/- 1788	10970 +/- 1749	14827 +/- 3056	12698 +/- 2257	0.4361	1.20	0.0006	1.63	0.0329	1.39
PC 36:1	27457 +/- 3847	37020 +/- 3078	33707 +/- 2569	27872 +/- 4138	0.0003	1.35	0.0126	1.23	0.9960	1.02
PC 36:2	119753 +/- 19192	173633 +/- 18761	151218 +/- 15994	119271 +/- 27247	0.0005	1.45	0.0421	1.26	1.0000	1.00
PC 37:4	10232 +/- 4024	14334 +/- 5030	25327 +/- 9110	23683 +/- 7323	0.6631	1.40	0.0017	2.48	0.0050	2.31
PC 38:2	14015 +/- 5585	15641 +/- 4747	15028 +/- 4167	14049 +/- 3478	0.9085	1.12	0.9753	1.07	1.0000	1.00
PC 38:3	11717 +/- 4591	16849 +/- 6096	17889 +/- 6499	17771 +/- 4776	0.3313	1.44	0.1884	1.53	0.2018	1.52
PC 40:10	34171 +/- 16982	50056 +/- 23218	49937 +/- 19778	47028 +/- 16507	0.4310	1.46	0.4374	1.46	0.6049	1.38
PE 12:0	390 +/- 114	556 +/- 161	402 +/- 71	424 +/- 68	0.0437	1.43	0.9966	1.03	0.9403	1.09
PE 32:1	1224 +/- 562	1036 +/- 499	1392 +/- 511	1095 +/- 449	0.8984	0.85	0.9241	1.14	0.9638	0.89
PE 34:1	1547 +/- 811	1313 +/- 686	1741 +/- 659	1362 +/- 596	0.9201	0.85	0.9530	1.12	0.9582	0.88
PE 34:2	1574 +/- 425	1310 +/- 413	2022 +/- 595	1603 +/- 435	0.7264	0.83	0.3098	1.28	0.9995	1.02
PE 36:1	676 +/- 420	601 +/- 365	784 +/- 404	649 +/- 334	0.9826	0.89	0.9521	1.16	0.9992	0.96
PE 36:2	1703 +/- 786	1433 +/- 681	2091 +/- 840	1649 +/- 701	0.9078	0.84	0.7724	1.23	0.9992	0.97
PE 38:2	712 +/- 371	596 +/- 345	748 +/- 344	619 +/- 272	0.9145	0.84	0.9971	1.05	0.9542	0.87

PE 38:3	717 +/- 268	623 +/- 309	705 +/- 246	696 +/- 199	0.9060	0.87	0.9998	0.98	0.9988	0.97
PE 38:4	1280 +/- 517	1181 +/- 524	1169 +/- 281	1210 +/- 275	0.9700	0.92	0.9586	0.91	0.9892	0.95
PG 34:1	442 +/- 89	343 +/- 108	515 +/- 84	421 +/- 37	0.1441	0.77	0.3839	1.16	0.9609	0.95
PG 36:1	401 +/- 78	307 +/- 61	437 +/- 35	384 +/- 57	0.0338	0.77	0.6782	1.09	0.9505	0.96
PG 36:2	437 +/- 128	388 +/- 172	509 +/- 164	412 +/- 120	0.9248	0.89	0.7971	1.17	0.9887	0.94
PI 32:1	1127 +/- 325	934 +/- 284	1051 +/- 308	861 +/- 170	0.5737	0.83	0.9553	0.93	0.3034	0.76
PI 34:0	462 +/- 170	378 +/- 145	430 +/- 155	384 +/- 95	0.6985	0.82	0.9736	0.93	0.7426	0.83
PI 34:1	2714 +/- 1105	2280 +/- 945	2635 +/- 983	2014 +/- 638	0.8213	0.84	0.9986	0.97	0.5105	0.74
PI 34:2	2000 +/- 423	1704 +/- 510	2375 +/- 697	1889 +/- 472	0.7305	0.85	0.5677	1.19	0.9798	0.94
PI 36:1	2384 +/- 1184	2015 +/- 949	2280 +/- 951	1681 +/- 606	0.8836	0.85	0.9969	0.96	0.5158	0.70
PI 36:2	2931 +/- 1172	2476 +/- 1029	2986 +/- 1167	2275 +/- 736	0.8456	0.84	0.9997	1.02	0.6447	0.78
PI 36:3	934 +/- 232	729 +/- 239	1384 +/- 537	1197 +/- 427	0.7484	0.78	0.1496	1.48	0.5786	1.28
PI 38:2	1481 +/- 633	1176 +/- 514	1609 +/- 734	1154 +/- 394	0.7633	0.79	0.9759	1.09	0.7233	0.78
PI 38:3	1403 +/- 452	1119 +/- 453	2099 +/- 1072	1475 +/- 557	0.8646	0.80	0.2522	1.50	0.9972	1.05
PI 38:4	435 +/- 128	343 +/- 123	754 +/- 351	569 +/- 188	0.8592	0.79	0.0524	1.73	0.6590	1.31
plasmenyl-PC 29:0	3207 +/- 411	3740 +/- 246	3578 +/- 641	3606 +/- 492	0.1753	1.17	0.4671	1.12	0.4034	1.12
plasmenyl-PC 30:0	6370 +/- 2063	5809 +/- 555	5439 +/- 636	5140 +/- 515	0.7954	0.91	0.4401	0.85	0.2115	0.81
plasmenyl-PC 32:0	32619 +/- 12075	29581 +/- 4208	27149 +/- 2297	22383 +/- 5674	0.8530	0.91	0.4858	0.83	0.0565	0.69
plasmenyl-PC 32:1	9360 +/- 2439	9573 +/- 1524	8694 +/- 1055	7895 +/- 924	0.9945	1.02	0.8632	0.93	0.3392	0.84
plasmenyl-PC 33:0	42110 +/- 15322	36423 +/- 5199	35031 +/- 2835	27339 +/- 6898	0.6363	0.86	0.4608	0.83	0.0235	0.65
plasmenyl-PC 34:0	28356 +/- 9367	28339 +/- 3663	26609 +/- 1959	22552 +/- 4806	1.0000	1.00	0.9379	0.94	0.2471	0.80
plasmenyl-PC 34:2	8098 +/- 1193	9652 +/- 1011	10689 +/- 2291	9444 +/- 1608	0.2914	1.19	0.0280	1.32	0.4134	1.17
plasmenyl-PC 36:0	13489 +/- 4473	15584 +/- 3686	12766 +/- 1355	12691 +/- 2140	0.6091	1.16	0.9732	0.95	0.9645	0.94
plasmenyl-PC 36:1	30323 +/- 8760	42474 +/- 10552	29305 +/- 6169	29359 +/- 7264	0.0541	1.40	0.9958	0.97	0.9964	0.97
plasmenyl-PC 37:0	14736 +/- 3656	16177 +/- 2310	17656 +/- 1561	15360 +/- 2064	0.7105	1.10	0.1610	1.20	0.9663	1.04
plasmenyl-PC 38:0	9873 +/- 3871	11650 +/- 3325	8817 +/- 1840	8780 +/- 2126	0.6685	1.18	0.9044	0.89	0.8952	0.89
plasmenyl-PC 38:1	29290 +/- 12975	38930 +/- 15639	28810 +/- 9279	27309 +/- 8927	0.4536	1.33	0.9999	0.98	0.9897	0.93
plasmenyl-PC 38:2	26641 +/- 12473	38722 +/- 15421	41483 +/- 16088	37789 +/- 12849	0.4078	1.45	0.2377	1.56	0.4767	1.42
plasmenyl-PC 38:4	8187 +/- 858	9652 +/- 948	12922 +/- 1519	11778 +/- 1692	0.1815	1.18	0.0002	1.58	0.0003	1.44
plasmenyl-PC 38:5	14818 +/- 1550	18158 +/- 1297	18400 +/- 2125	16137 +/- 1520	0.0048	1.23	0.0025	1.24	0.4565	1.09
plasmenyl-PE 32:1	527 +/- 154	441 +/- 126	549 +/- 124	426 +/- 68	0.5601	0.84	0.9853	1.04	0.4273	0.81
plasmenyl-PE 34:2	7320 +/- 988	8162 +/- 759	8046 +/- 518	7471 +/- 693	0.1889	1.12	0.3021	1.10	0.9818	1.02
PS 32:0	700 +/- 230	537 +/- 161	624 +/- 177	567 +/- 164	0.3698	0.77	0.8654	0.89	0.5439	0.81
PS 32:1	1331 +/- 393	1075 +/- 168	996 +/- 214	813 +/- 107	0.2320	0.81	0.0754	0.75	0.0031	0.61
PS 34:0	3665 +/- 930	2718 +/- 699	3265 +/- 551	2711 +/- 599	0.0863	0.74	0.7188	0.89	0.0831	0.74
PS 34:1	1855 +/- 401	1477 +/- 240	1903 +/- 267	1454 +/- 246	0.1052	0.80	0.9904	1.03	0.0794	0.78
PS 36:1	3335 +/- 619	2525 +/- 571	3929 +/- 572	3235 +/- 782	0.1125	0.76	0.3304	1.18	0.9911	0.97
PS 36:2	1248 +/- 296	1012 +/- 220	1511 +/- 337	1143 +/- 248	0.4060	0.81	0.3149	1.21	0.8944	0.92
PS 38:2	1009 +/- 244	731 +/- 110	1405 +/- 229	1162 +/- 278	0.1217	0.72	0.0150	1.39	0.5847	1.15
PS 39:8	5725 +/- 772	4204 +/- 1020	5712 +/- 728	4694 +/- 865	0.0139	0.73	1.0000	1.00	0.1361	0.82

PS 40:2	1005 +/- 161	712 +/- 98	1267 +/- 190	1042 +/- 242	0.0272	0.71	0.0549	1.26	0.9807	1.04
SM 34:0	11261 +/- 3762	9591 +/- 1242	10426 +/- 1195	8737 +/- 1493	0.4992	0.85	0.8921	0.93	0.1673	0.78
SM 34:1	8652 +/- 3842	6422 +/- 693	7533 +/- 427	6998 +/- 1379	0.2147	0.74	0.7478	0.87	0.4606	0.81
SM 34:2	3440 +/- 808	3501 +/- 337	3334 +/- 367	3608 +/- 839	0.9980	1.02	0.9891	0.97	0.9594	1.05
SM 35:0	12660 +/- 4500	11518 +/- 1784	10492 +/- 935	8582 +/- 1838	0.8481	0.91	0.4293	0.83	0.0369	0.68
SM 36:1	7348 +/- 1773	8135 +/- 1624	8273 +/- 1126	7096 +/- 987	0.7285	1.11	0.6195	1.13	0.9871	0.97
SM 37:1	11087 +/- 3043	13723 +/- 3166	10101 +/- 1151	10086 +/- 2382	0.2450	1.24	0.8883	0.91	0.8838	0.91
SM 38:0	14457 +/- 5312	13190 +/- 1671	14512 +/- 1044	11907 +/- 2366	0.8664	0.91	1.0000	1.00	0.4227	0.82
SM 39:1	15142 +/- 4294	20657 +/- 5541	14174 +/- 2548	14628 +/- 3645	0.0880	1.36	0.9716	0.94	0.9956	0.97
SM 42:2	9026 +/- 2904	10875 +/- 3159	11586 +/- 3385	11079 +/- 2965	0.6855	1.20	0.4305	1.28	0.6114	1.23
TG 50:2	11198 +/- 1687	13591 +/- 4028	10455 +/- 1239	11341 +/- 2162	0.3067	1.21	0.9450	0.93	0.9996	1.01
TG 52:3	9711 +/- 1165	11505 +/- 1493	9035 +/- 1222	8604 +/- 1215	0.0669	1.18	0.7578	0.93	0.3879	0.89
TG 52:4	6030 +/- 2113	8644 +/- 3342	18690 +/- 7804	18063 +/- 6761	0.8128	1.43	0.0015	3.10	0.0024	3.00
TG 54:2	7063 +/- 2605	9423 +/- 2998	8655 +/- 2335	9358 +/- 1980	0.3160	1.33	0.6400	1.23	0.3393	1.33
TG 54:4	4714 +/- 1594	6660 +/- 2871	11545 +/- 4738	11597 +/- 4137	0.7361	1.41	0.0074	2.45	0.0069	2.46

Intensities were normalized by class and significant differences were assessed by one-way ANOVA with post hoc Dunnett's correction

Table ST3. Primer Sequences Used

Name	Primer Sequence
hEGR1_Forward	ATGTGCAATTGTGAGGGACAT
hEGR1_Reverse	CAGCTCAGCCCTCTTCCTTAT
hEGR2_Forward	CAATAGGTTGGGAGATGCTGA
hEGR2_Reverse	CAAATCAGTCCCAAGCCATAA
hATF3_Forward	CAAGATATTCAGGTGGCCAGA
hATF3_Reverse	GAGTTTGCAACAGAGGACCTG
hIL20_Forward	GGGAGGAAGCAATGAAGAAAT
hIL20_Reverse	AATGTCTAGTTCCCCCAAAGC
hTNF_Forward	CTTTGATCCCTGACATCTGGA
hTNF_Reverse	AGGCCTAAGGTCCACTTGTGT
hCREBRF_Forward	AGAGAAGAGTGGAAGGGCAAG
hCREBRF_Reverse	GGATCCTACGCAACTATGCAA

Table ST4: iRegulon Output for Genes Upregulated by Statin

# Rank	Motif id	AUC	NES	ClusterCode	Transcription factor
1	jaspar-MA0356.1	0.0828617	5.13456	1	
2	transfac_pro-M01897	0.0828617	5.13456	1	
3	yetfasco-505	0.0828617	5.13456	1	
4	transfac_pro-M01007	0.0806247	4.94018	2	SRF
5	flyfactorsurvey-HLH106_SANGER_5_3_FBgn0261283	0.0782434	4.73326	3	SREBF1,SREBF2
6	transfac_pro-M00392	0.0742333	4.38482	2	MEF2A,SRF,MEF2B
7	transfac_pro-M01304	0.0739034	4.35616	2	SRF
8	transfac_pro-M00395	0.0730478	4.28181	4	HOXA3
9	flyfactorsurvey-dl_FlyReg_FBgn0000462	0.0730272	4.28002	5	RELB
10	jaspar-MA0083.1	0.0723983	4.22538	2	SRF
11	hdpi-RARB	0.072254	4.21284	6	RARB
12	transfac_pro-M01164	0.0706562	4.074	2	
13	transfac_pro-M01257	0.0696356	3.98532	2	SRF
14	jaspar-MF0008.1	0.0695944	3.98174	2	SRF
15	transfac_pro-M01002	0.0695944	3.98174	5	DEAF1
16	selexconsensus-Rel	0.0694294	3.96741	5	NFKB1,NFKB2
17	stark-GGGGAMWWCCM	0.0692129	3.9486	5	HIVEP3,HIVEP1,HIVEP2,ZNF831
18	transfac_pro-M00810	0.0689861	3.92889	2	SRF
19	transfac_pro-M00922	0.0683264	3.87156	2	SRF
20	hdpi-DGCR8	0.0682748	3.86709	7	DGCR8
21	transfac_pro-M00774	0.0664914	3.71212	5	NFKB1,RELA,NFKB2
22	flyfactorsurvey-H2.0_Cell_FBgn0001170	0.064708	3.55716	1	HLX
23	transfac_pro-M00186	0.064708	3.55716	2	SRF
24	jaspar-MA0298.1	0.0646977	3.55626	8	ZNF22,EGR1,EGR2,WT1,EGR3,EGR4
25	hdpi-FAM119B	0.0646358	3.55089	9	METTL21B
26	transfac_pro-M01061	0.0645637	3.54462	2	SRF,MEF2A,MEF2B
27	hdpi-FOXP4	0.064007	3.49625	5	FOXP4
28	transfac_pro-M00109	0.063873	3.48461	10	CEBPB,CEBPE,CEBPG,CEBPA,CEBPD
29	jaspar-PB0105.1	0.0638318	3.48102	4	ARID3A
30	transfac_pro-M00445	0.0638111	3.47923	4	
31	transfac_pro-M00159	0.0636256	3.46311	10	CEBPA
32	transfac_pro-M00749	0.0635019	3.45236	3	SREBF1
33	jaspar-MA0105.1	0.0634503	3.44788	5	NFKB1
34	flyfactorsurvey-lola-PC_SANGER_5_FBgn0005630	0.06344	3.44698	5	
35	transfac_pro-M01223	0.0633988	3.4434	5	NFKB1,RELA,NFKB2
36	jaspar-MA0001.1	0.0630586	3.41384	2	
37	transfac_pro-M00394	0.0629761	3.40668	2	MSX1
38	transfac_pro-M00776	0.0628112	3.39234	3	SREBF2,SREBF1
39	transfac_pro-M00101	0.0625122	3.36637	1	
40	hdpi-EVX1	0.0624195	3.35831	7	EVX1
41	transfac_pro-M00640	0.0623782	3.35472	1	HOXA4
42	transfac_pro-M00393	0.0621824	3.3377	2	SRF,MEF2B
43	transfac_pro-M01064	0.0621824	3.3377	2	SRF,MEF2B
44	transfac_pro-M00713	0.0620277	3.32427	4	TBP,TBPL2,TBPL1
45	jaspar-PB0078.1	0.0618319	3.30725	2	SRF
46	hdpi-SMAD2	0.0616875	3.29471	2	SMAD2
47	transfac_pro-M00949	0.0615741	3.28486	2	MEF2A,MEF2B
48	transfac_pro-M01869	0.0614814	3.27679	10	CEBPG,CEBPB,CEBPD,CEBPA
49	transfac_pro-M00215	0.0612958	3.26067	2	SRF
50	jaspar-PB0128.1	0.0612443	3.25619	4	GCM1
51	transfac_pro-M00054	0.0612133	3.2535	5	NFKB1,RELA,NFKB2,REL
52	jaspar-MA0022.1	0.061203	3.25261	5	RELA,REL,RELB
53	jaspar-MA0108.2	0.0610175	3.23649	4	TBP,TBPL2
54	transfac_pro-M00252	0.0610175	3.23649	4	TBP,TBPL2
55	jaspar-MA0108.1	0.0609144	3.22753	4	TBP,TBPL2
56	jaspar-MA0430.1	0.0608835	3.22484	11	
57	yetfasco-589	0.0608835	3.22484	11	
58	hdpi-IVD	0.060801	3.21768	12	IVD
59	jaspar-PB0184.1	0.0606979	3.20872	4	TBP
60	transfac_pro-M00216	0.0600175	3.1496	6	TBP
61	jaspar-MA0061.1	0.0595639	3.11019	5	NFKB1,RELA,NFKB2,REL
62	yetfasco-1874	0.0595021	3.10481	13	
63	elemento-CTTATCAG	0.0591516	3.07436	8	GATA5
64	transfac_pro-M01809	0.0590794	3.06809	2	
65	elemento-CCTTATC	0.0590485	3.0654	14	GATA5
66	jaspar-PF0121.1	0.05843	3.01166	2	SRF
67	hdpi-PHLDA2	0.0584197	3.01076	7	PHLDA2

Supplemental methods:

MTS assay

The effect of simvastatin and lysoPC 18:1 on HBE1 cell viability was measured by MTS assay. Briefly, 10,000 HBE1 cells per well were seeded in a 96-well microplate. After 24 hours treatment of 1µM simvastatin and/or 40nM lysoPC18:1, cells were treated with pneumolysin for 4 hours. The medium was then replaced with freshly prepared medium containing MTS (Promega) at a 1:5 dilution (100 µL per well). The cells were incubated for 60 minute and the absorbance at 490 nm was measured with the EnVision multi-label plate reader.
